# The Associations of Affection and Rejection During Adolescence with Interpersonal Functioning in Young Adulthood: A Macro- and Micro- Level Investigation Using the TRAILS TRANS-ID Study

**DOI:** 10.1007/s10964-022-01660-y

**Published:** 2022-07-19

**Authors:** Larisa Morosan, Johanna T. W. Wigman, Robin N. Groen, Marieke J. Schreuder, Marieke Wichers, Catharina A. Hartman

**Affiliations:** University Medical Center Groningen, ICPE, University of Groningen, Groningen, The Netherlands

## Abstract

Affection and rejection in close relationships during adolescence are thought to impact adult interpersonal functioning, but few studies focused on how the quality of adolescents’ relationships with different people (e.g. parents, peers, and teachers) impacts the daily, micro-level social experiences as well as general, macro-level interpersonal functioning in young adulthood. The present study investigated the associations between: (i) parental, teacher and peer affection and rejection during adolescence and macro-level (over several months) interpersonal functioning as well as different patterns (i.e. mean, variability and inertia) of micro-level (daily social experiences) during young adulthood; (ii) macro-level interpersonal functioning and the patterns of micro-level social experiences during young adulthood. The sample consisted of N = 122 (43% female) youth. At 11.2 ± 0.4 and 16.0 ± 0.6 years old, self- and other-reported parental, peer and teacher affection and rejection were assessed. At 23.7 ± 0.6 years old, participants reported daily social experiences and interpersonal functioning across six months. The results suggested that: (i) higher teacher-reported peer rejection was associated with lower macro-level interpersonal functioning, higher means and higher variability in negative social experiences during adulthood; (ii) higher macro-level interpersonal functioning during young adulthood was associated with higher means and lower inertia in positive and lower variability in negative daily social experiences. These findings indicate that the affection and rejection during adolescence impact interpersonal functioning at macro- and micro-level during adulthood. The present study also shows distinct associations between macro-level interpersonal functioning and dynamics in daily social experiences.

## Introduction

Human development is embedded in interpersonal contexts, which shape the way individuals represent themselves and others (Lamblin et al., 2017). These representations translate into everyday social behaviors and experiences. Adolescence is a critical period for psychosocial and identity development (Sebastian et al., 2008) and affection and rejection during this period impacts interpersonal functioning and interpersonal problems during adulthood (Boisvert and Poulin 2016). However, most of the research has focused on parental and peer affection and rejection (e.g. Richards et al., 2019), not taking into consideration adolescents’ relationships with other important people, such as teachers. Investigating the simultaneous impact of parental, peer, and teacher affection and rejection can reveal to what extent the relationships during adolescence with each of these people impact future interpersonal functioning. Furthermore, prior research assessed interpersonal functioning mostly at the macro-level (i.e. across months or years). However, social relationships happen at a micro-level, meaning at shorter time intervals (i.e. across hours or days). Dynamic patterns of daily social experiences can inform about the individual adjustment to everyday social contexts (Houben et al., 2015). Investigating the associations among affection and rejection during adolescence and macro- and micro-level interpersonal functioning during adulthood can provide additional insights about the mechanisms underpinning social development from adolescence to adulthood. Finally, little is known about how micro-level patterns of daily social experiences and macro-level interpersonal functioning are associated. Addressing these gaps in the literature, the first aim of this study is to investigate the relations between self- and other-reported parental, peer and teacher affection and rejection during adolescence and adult interpersonal functioning, assessed both at the macro- (across several months) and micro- level (daily social experiences). A secondary aim of the study is to understand which dynamic patterns of daily social experiences (i.e. mean, inertia, variability of daily social experiences) are associated with macro-level interpersonal functioning during young adulthood.

Adolescence represents a critical period for social development (Andrews et al., 2021). During adolescence, self-consciousness and social awareness develop, leading to increased sensitivity to social relationships (Kilford et al., 2016). Positive relationships are described by affectionate and sensitive responses to youth’s needs and emotions, whereas negative relationships are described by rejection, defined as insensitive emotional responses (Lorijn et al., 2021). Many authors have highlighted the role of parental affection and rejection in shaping the mental representations about self and others, which impacts future interpersonal functioning (e.g. Fonagy et al., 2002). Other studies have indicated that parental affection is associated with a stable sense of the self and positive world-view (e.g. McAdams et al., 2017), whereas parental rejection is associated with negative self and others representations as well as emotional instability (e.g. Rohner & Lansford, 2017). Furthermore, the quality of relationships with parents represents the basis for future relationships, for example with friends and romantic partners (Meeus, 2016).

During adolescence, social contexts diversify and the relationships with people outside of family, such as peers, gain in importance. Compared to other developmental periods (e.g. childhood and adulthood), positive relationships with peers during adolescence are more rewarding, while negative social experiences, such as peer rejection, generate stronger negative affective reactions (Silk et al., 2012). The increased importance of peer relationships during adolescence enhances the impact of both peer rejection and affection on future interpersonal development (Marion et al., 2013). Besides time spent with parents and peers, youth spend a substantial amount of time at school. Teachers thus represent additional important figures in their lives (e.g. Valiente et al., 2020). In line with this, perceived teacher affection has been associated with better interpersonal functioning (e.g. Breeman et al., 2015). Although the importance of parental, peer and teacher affection and rejection during adolescence for the interpersonal functioning during adulthood each has been highlighted (e.g. Rohner & Lansford, 2017), there is a lack of studies investigating the *simultaneous* impact affection and rejection in different interpersonal contexts (with parents, peers and teachers) on adult interpersonal functioning.

Another gap in the literature is that most of the previous studies have focused on interpersonal functioning at the macro-level, i.e., asking individuals to recall their social experiences over longer periods of time (months or years). However, social interactions take place at shorter time intervals (minutes, hours, days), which are not always captured optimally by macro-level assessments (Gable et al., 2012). At the micro-level, interpersonal functioning has been studied using intensive longitudinal designs (ILD), collecting repeated measures of social experiences in everyday life situations. For this reason, ILD are believed to provide measures of interpersonal functioning that are ecologically valid, giving access to real-life aspects of social experiences (Gable et al., 2012). In addition, ILD yield a large number of assessments for each individual, allowing the investigation of within-person processes captured by dynamic patterns of daily social experiences. As social experiences fluctuate depending on external contexts and individuals’ internal regulatory strategies, dynamics in daily social experiences might represent individuals’ tendencies of responding to diverse social contexts (e.g. Ebner-Priemer et al., 2009). Thus, to gain a better understanding about interpersonal functioning, research needs to extend beyond the macro-level assessments and incorporate real-life, micro-level social experiences.

**Fig. 1:**
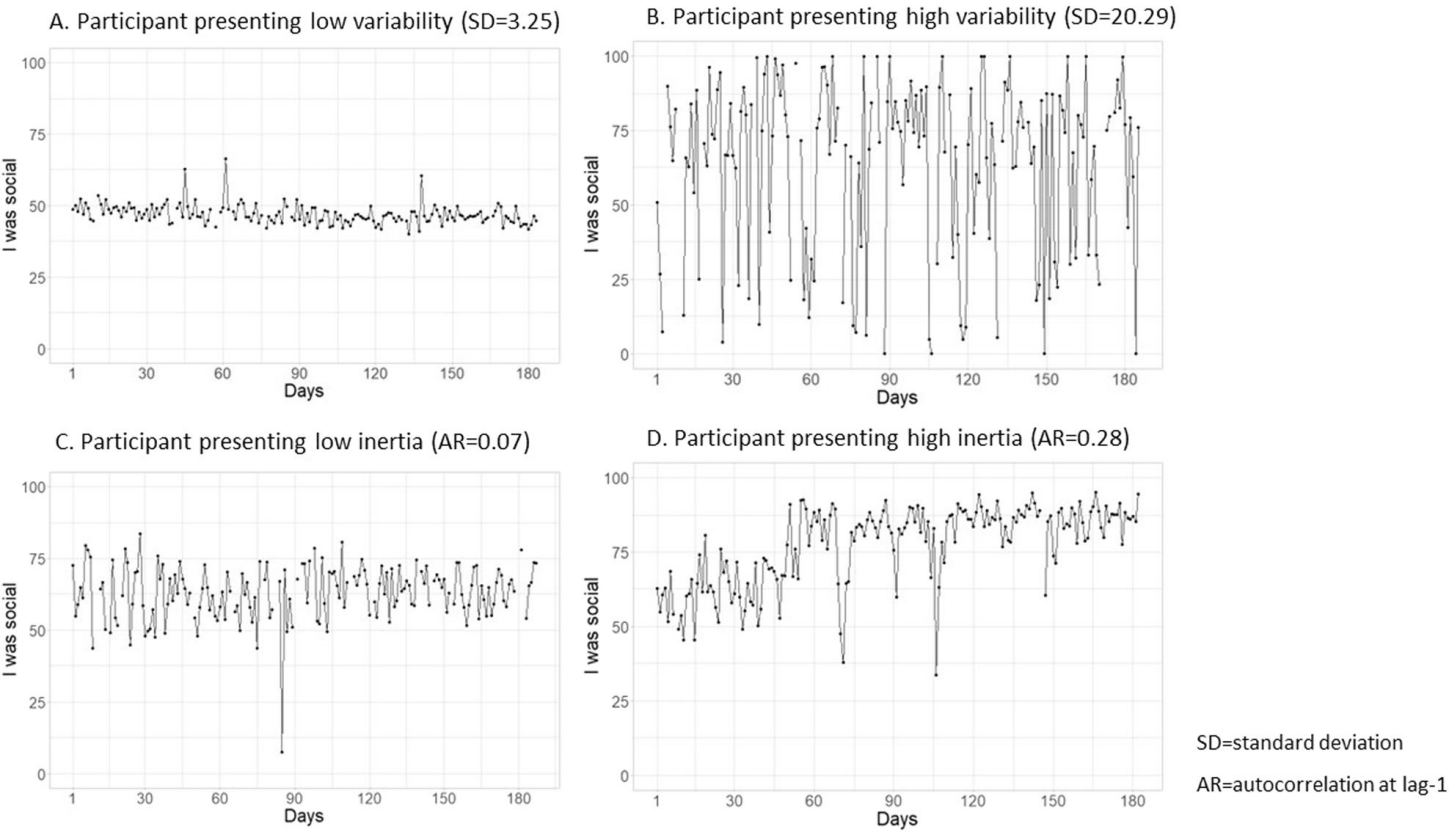
Illustration of high and low variability and inertia in the item “I was social”, using the data from four participants in the study, each presented in a separate panel. **A**, **B** Participants with high and low variability and **C**, **D** participants with high and low inertia. In each panel, the *x* axes present the number of days in the study, the *y* axes present the daily scores of the item, ranging from 0 to 100

Different indices, such as variability and inertia, have been used to study these dynamic patterns of daily social experiences measured using ILD. Variability describes the overall fluctuations in daily mental states, usually operationalized in terms of within-person variance or within-person standard deviation (Wichers et al., 2010). Some variability in daily social experiences is considered a normative adaptation to different social contexts (Kaurin et al., 2021), but increased variability describes the tendency to experience very intense and irregular reactions to social environments (Houben et al., 2015). For example, a person with high variability in how social they are may be very social one day and not at all on the next day. Inertia describes the persistence of mental states from one moment or day to another, which is measured as the autocorrelation of mental states over time. A high autocorrelation indicates that an affective state at one time point is highly predictive of the mental state at the next time point and is thought to represent rigidity of emotional responses (Kuppens et al., 2010). High social inertia has been described as the tendency to have a specific social experience for several consecutive days (Elmer et al., 2020). For example, high inertia in sociability may involve being very social over several consecutive days. As an illustration, Fig. 1 shows patterns of high and low inertia and variability in the item “I was social “ of four young adults taking part in the present study.

Increased variability and inertia in daily social experiences have been linked to different patterns of responding to social contexts (e.g. Ringwald et al., 2021), but little is known about what predicts these different dynamic patterns of social experiences. Recent research has focused on the associations between macro-level, retrospective assessments of parent-adolescent relationships and daily social experiences during adolescence and young adulthood measured using ILD (Achterhof et al., 2022). This study shows that positive parenting practices are associated with higher positive and lower negative daily social experiences, whereas negative parenting practices are associated with higher daily negative social experiences and lower positive social experiences. Although these results shed some light on the cross-sectional relations between the perceived parenting practices and the mean level of daily social experiences during adulthood and adolescence, little is known about the potential long-term impact of the interpersonal affection and rejection during adolescence on the daily social experiences during adulthood. Furthermore, these studies focused only on the mean level of daily social experiences. Several authors have suggested that poor relationship quality during childhood and adolescence is associated with individuals’ inability to adapt to changing social environments or an unstable self-concept (e.g. Fonagy et al., 2002). As inertia and variability in daily social experiences reflect individuals’ tendencies to respond to daily social contexts (i.e. increased rigidity or variability in social experiences), investigating the associations between these dynamic patterns of daily social experiences and interpersonal affection and rejection during adolescence might shed light on the potential long-term effects of the quality of different social relationships on the daily interpersonal functioning, as pursued in this study.

Although relations between macro- and micro-level assessments of interpersonal functioning have been hypothesized by several authors (e.g. Wichers, 2014), the literature has so far not sufficiently addressed which specific aspects of daily social experiences are associated with macro-level interpersonal functioning. Most previous studies have focused on the mean level of daily social experiences, averaged across several weeks and months (e.g. Schneider et al., 2017), suggesting that the type and intensity of daily social experiences are reflected, to a certain extent, into macro-level reports of interpersonal functioning. Additionally, one study suggests that macro-level reported satisfaction and trust in romantic relationships are associated with greater variability in daily social experiences (Campbell et al., 2010). However, there is a clear need for a better understanding which specific dynamic patterns of daily social experiences are linked to interpersonal functioning.

## The current study

**Fig. 2:**
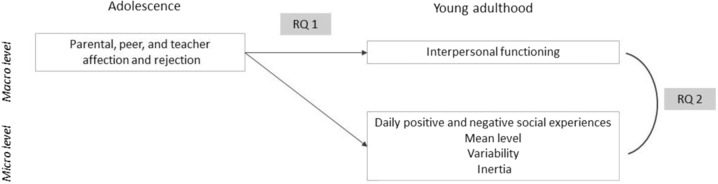
A schematic representation of the research questions (RQ) of the present study

Affection and rejection during adolescence have been previously identified as important for interpersonal functioning during adulthood. Nevertheless, little is known about the simultaneous impact of parental, peer, and teacher affection and rejection during adolescence on social experiences during adulthood. The first aim of the present study is to investigate the longitudinal associations between self- and other-reported parental, peer, and teacher affection and rejection during adolescence and interpersonal functioning measured at both macro- (across several months) and micro- (daily) level during young adulthood (Research Question 1). Higher parental, peer and teacher affection during adolescence is expected to be associated with better interpersonal functioning, whereas higher rejection is expected to be associated with lower interpersonal functioning assessed at both macro- and micro-level. As a secondary aim, cross-sectional associations between macro- and micro-level assessments of interpersonal functioning are investigated (Research Question 2), so that we get a better understanding on how these levels of interpersonal functioning are linked. Figure 2 presents the two research questions of the present study.

## Methods

### Sample and procedure

Data were used from the TRAILS TRANS-ID study, a 6 months daily diary study aiming to explore the daily dynamics in mental states in a group of at-risk youth. The participants were recruited from the ongoing TRAILS-CC (Tracking Adolescents’ Lives Survey Clinical Cohort) study that follows a cohort of 543 at-risk youth who consulted the child psychiatric clinic at the University Medical Center Groningen at least once before the age of 11 years (for a detailed presentation of the TRAILS cohort, see Huisman et al., 2008; Oldehinkel et al., 2015). In the present study the first (T1- at the age of 11.19 ± 0.4 years old) and third (T3- at the age of 15.96 ± 0.6 years old) assessments of the TRAILS-CC study were used.

**Fig. 3:**
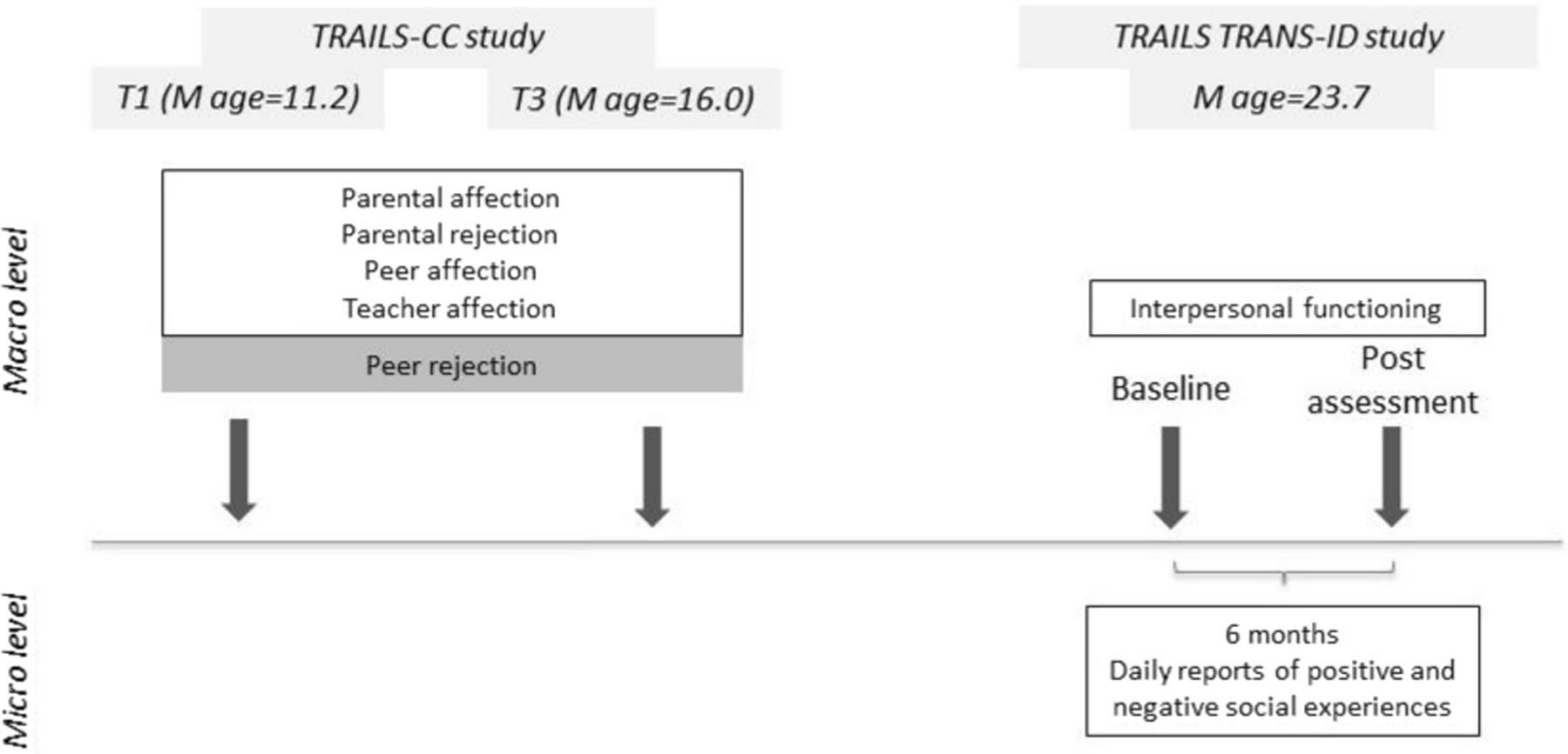
The study design and the variables measured at each time point that were included in the present study. The variables measured using self-reports are presented in white squares and those assessed using other-reported questionnaires are presented in grey

Between T5 and T6 of TRAILS-CC (at the age 23.64 ± 0.6 years old), 134 youth (76 males) who completed at least one assessment in the TRAILS-CC study were enrolled in the TRAILS TRANS-ID daily diary study (for a detailed description of the study, see Schreuder et al., 2020). The daily diary assessments took place between December 2017 and September 2018. Each evening, at a fixed time at their own convenience, the participants received a text message with a link to an electronic diary including questions referring to their different experiences (e.g. emotions, thoughts, events, social interactions) during the past day. The time of completing the diary differed among participants, but the interval between two consecutive assessments was 24 h for each person. The participants had 3 h to complete their diaries. Out of 134 participants agreeing to take part in the study, 12 dropped out, resulting in a final sample of 122 participants (53 females). The youth who dropped out during the diary period did not differ from those who remained in the study regarding their baseline interpersonal functioning scores (*t*(132) = −0.43, *p* = 0.664), age (*t*(132) = −0.80, *p* = 0.969), sex (*χ2*(1) < 0.01, *p* = 0.90), or any variables of interest measured at either T1 or T3 in the TRAILS-CC study (*p* > 0.068). The average number of days in the diary study were 183.9 (ranging from 177 to 225 days). On average, the participants completed 162.3 diaries (88% of diaries), resulting in 19806 complete daily diaries across all participants. The diaries were administered and stored via Roqua (www.roqua.nl). Before and after the 6 months diary assessment, clinical interviews were conducted by two of the co-authors (R.N.G. and M. J. S.) and a research assistant under their supervision. Figure 3 presents the assessments from the TRAILS-CC and TRAILS TRANS-ID included in the present study.

Regarding the mental health problems of the participants in the TRAILS-TRANS-ID study, recent investigations show that the youth who completed the daily diary do not significantly differ from the TRAILS-CC participants who did not participate in the daily diary study in their rates of mental health problems during adolescence (Groen et al., 2021, submitted). However, they reported significantly higher mental health problems compared to the general population sample (Groen et al., 2021, submitted). In addition to substantially enhanced mental health problems, participants in the TRAILS-CC cohort also presented with several DSM-IV diagnosis as based on the World Mental Health Organization Composite Diagnostic Interview (CIDI; Kessler & Üstün, 2004): substance abuse (25.5%), social phobia (21.8%), major depression disorder (20.1%), attention deficit/hyperactivity disorder (ADHD) (14%), oppositional defiant disorder (18.6%) (for a full description of the sample psychiatric diagnoses, see Oldehinkel et al., 2015). Before and after the participation in the diary study, diagnostic criteria for some psychiatric diagnoses were measured using mini-SCAN clinical interview (Nienhuis et al., 2010). The most prevalent psychiatric diagnoses were mood disorders (20% at the baseline and 19% at post daily diary assessment) and anxiety disorders (10% at the baseline and 7% at the post-assessment). Participants also met the clinical diagnostic criteria for ADHD (6% at baseline and 7% at post-assessment), psychotic disorders (1% at baseline and 4% at post-assessment), substance use disorder (2% baseline and 2% at the post-assessment) and adjustment disorder (1% at baseline)(for detailed information on the sample characteristics of the TRAILS-TRANS-ID study, see Schreuder et al., 2020). In all, the study sample should be seen as a high-risk sample, based on being referred for early childhood psychopathology and current high symptoms levels which, for different persons and at different timepoints and dependent on the diagnostic instrument, may qualify for a clinical diagnosis.

### Measures

#### Self-reported parental affection

At T1, perceived parental affection and rejection were assessed using 7 items from the ‘emotional warmth’ (e.g. “When things are going badly for you, is your father / mother trying to comfort or help you?”) subscale of the Egna Minnen Beträffande Uppfostran (My Memories of Upbringing for Children-EMBU-C; Markus et al., 2003). At T3, perceived parental affection was assessed using 7 items from the ‘problem-solving’ and ‘parental solicitation’ subscales (e.g. “Your father/mother tries to understand how you thought and felt”) of Parental Reactions to Child Behaviors questions (based on Tilton-Weaver et al., 2010). The answers referring to relationships with fathers or mothers were highly correlated (*r* = 0.51–0.74 for EMBU-C and *r* = 0.51–0.80 for Parental Reactions items) and therefore combined into one mean score describing overall parental warmth. Internal consistency of the averaged items was α = 0.80 (at T1) and α = 0.79 (at T3). Tables S1 and S2 from the Supplementary Material present all the items used in the present study, reliability information, and the model fit indices of the CFA testing the measurement model.

#### Self-reported parental rejection

At T1, perceived parental rejection was assessed using 5 items from the ‘rejection and overprotection’ (e.g.” Does your father/ mother blame you for everything?”) subscales of the Egna Minnen Beträffande Uppfostran (My Memories of Upbringing for Children-EMBU-C; Markus et al., 2003). At T3, parental rejection were assessed using 5 items from ‘guilt inducing’ and ‘angry outbursts’ subscales (e.g.” Your father/mother is quiet and cold to you”) of Parental Reactions to Child Behaviors questions (based on Tilton-Weaver et al., 2010). The answers for both parents were highly correlated (*r* = 0.46–0.75 for EMBU-C and *r* = 0.51–0.79 for Parental Reactions) and therefore averaged. Internal consistency of the averaged items was α = 0.68 (at T1) and α = 0.73 (at T3). The selected items are presented in the Supplementary Materials, in Table S2.

#### Self-reported teacher affection

Teacher affection at T1 and T3 was assessed using the Social Production Function Questionnaire (SPF; Ormel et al., 1997). The items referred to perceived affection and behavioral confirmation received from teachers (e.g. “Most teachers take my feelings into account”). Internal consistency was α = 0.85 at both T1 and T3.

#### Self-reported peer affection

Peer affection was assessed at T1 and T3 using the Social Production Function Questionnaire (SPF; Ormel et al., 1997). The items referred to perceived affection and behavioral confirmation from classmates (e.g. “Most classmates like to do things with me.”). Internal consistency was α = 0.90 at T1 and T3.

#### Parent-reported peer rejection

Peer rejection was reported by parents at T1 and T3 using four items from the social problems subscale from CBCL (e.g. “Other boys/girls do not like him/her”) from the Child Behavior Checklist (CBCL) (Achenbach, 2001). Internal consistency was α = 0.78 (at T1) and α = 0.77 (at T3).

#### Teacher-reported peer rejection

Peer rejection was reported by teachers at T1 and T3 using the four items from the social problems subscale from TRF (e.g. “Other boys/girls do not like him/her”) Teacher’s Report Form (TRF) (Achenbach, 2001). The same items were used for the parent and teacher reported peer rejection. Internal consistency was α = 0.86 (at T1) and α = 0.78 (at T3).

#### Interpersonal functioning at micro-level

Six items describing daily social experiences (3 items describing positive social experiences: ‘I was at ease with others’, ‘I felt that others liked me’, ‘I was social’, and 3 items describing negative social experiences: ‘I had a fight’, ‘I felt that others were annoyed by me’, ‘I felt lonely’) were selected from the daily diary protocol from the TRAILS-TRANS-ID study. Participants had to rate the extent to which the item referred to what happened during the past day on a visual analogue scale (VAS) ranging from 0 to 100.

#### Interpersonal functioning at macro-level

The Groningen Social Behavior Questionnaire (GVSG; Jong & Lubbe, 2001) was used to assess interpersonal functioning in different social contexts (with family, friends, children, partner, work and education) at baseline and post daily diary period (e.g. “I got along well with my parents”, “I enjoyed spending time with my friends and/or good acquaintances”). Few participants indicated having children (*n* = 10), thus the subscales referring to the relationships with children were not included in the present study. Items related to non-social aspects (e.g. performance at work/education) from the ‘work’ and ‘education’ subscales were also not included. Two mean scores (one for the baseline and one for the post daily diary assessment) including all the items referring to the relationship with family, friends, partner and colleagues were computed. The negative items were recoded so that higher mean scores indicate better interpersonal functioning. Internal consistency was α = 0.70 (baseline) and α = 0.68 (post diary assessment). Table S3 in the Supplementary Materials presents the selected items and the reliability information.

#### Socio-economic status

The socio-economic status (SES) of the participants’ family was measured at T1 using five indicators: family income, education and occupation status of both mother and father. Education level of the parents ranged from elementary education to university degree and occupation level was based on the International Standard Classification of Occupations (Ganzeboom & Treiman, 1996). Family incomes ranged from less than 1500 euros (approx.. 1600 US dollars) per month to higher than 8500 euros (approx. 8900 US dollars) per month. A mean score describing the SES status was computed after each of the five indicators scores was standardized: SES = mean(z score occupation father, z score occupation mother, z score education father, z score education mother, z score income family).

#### IQ

The deviation quotient, a proxy for intelligence quotient, was assessed using the Vocabulary and Block Design subtests of Weschler Intelligence Scale for children (WISC) at T1 (Vandersteene et al., 1986)

### Statistical analysis

**Fig. 4:**
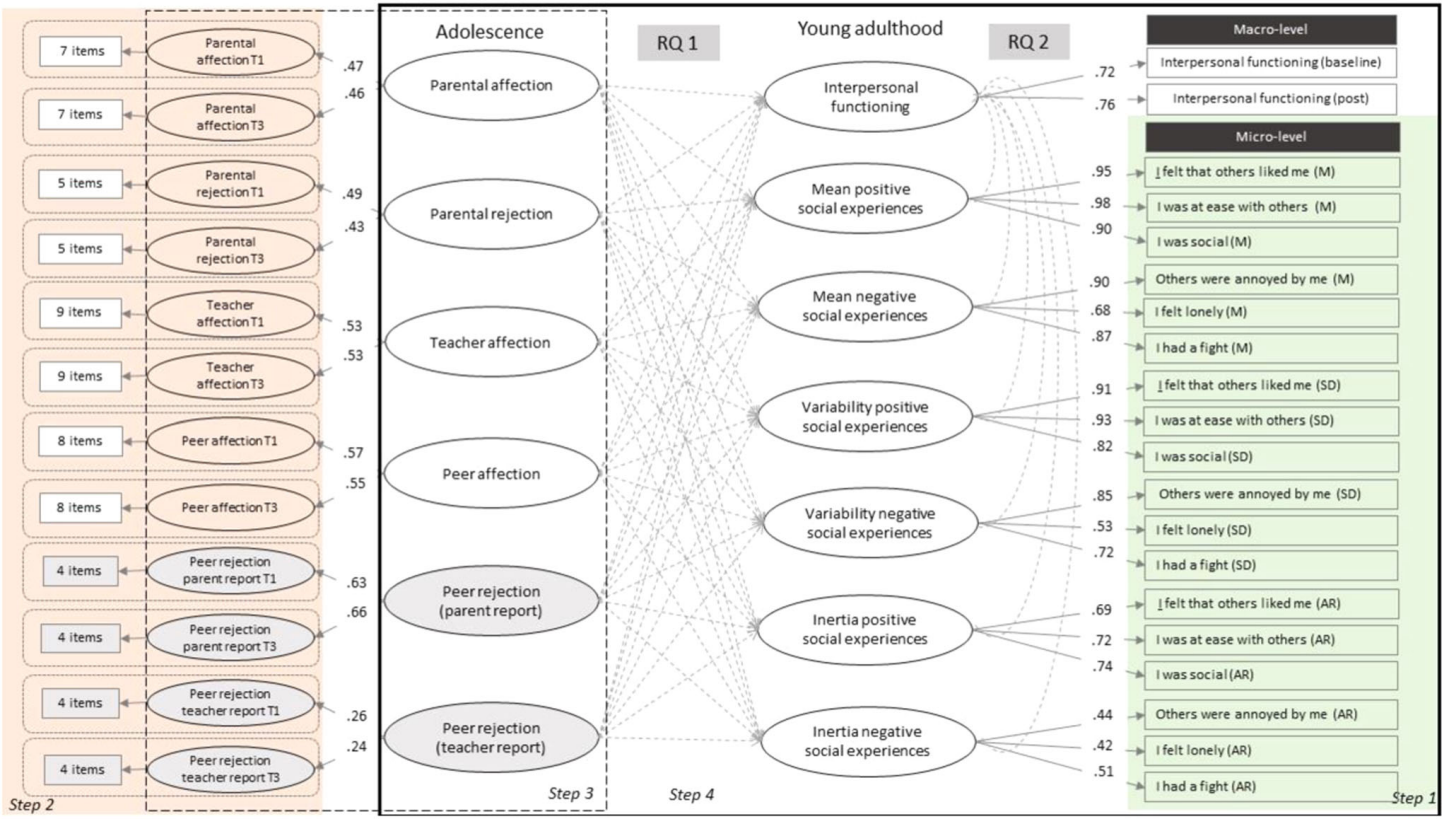
Overview of the steps taken for the statistical analysis. Step 1-in green square: Computing the indices (mean-M, standard deviation-SD, and inertia-AR) for each item describing daily social experiences; Step 2-in orange square: Factor score extraction from one-factor CFAs in which item scores selected for the self-reported (in white) and other-reported (in grey) parental, teacher, peer affection and rejection at T1 and T3 in the TRAILS-CC study were fitted into 12 latent factors (the factor loadings for the latent factors fitted in Step 2 are discussed in the Supplementary materials in the Measures section); Step 3- in dotted square: Factor score extraction from one CFA in which the factor scores extracted in Step 1 were into fitting into 6 latent factors describing the self-reported (in white) and other-reported (in grey) parental, teacher, peer affection and rejection, across adolescence; Step 4-in solid black square: Exploring the research questions (RQ 1 and RQ 2) using one SEM. For RQ1, the factor scores extracted in Step 3 were used in the SEM as predictors for seven latent factors describing interpersonal functioning during adulthood (all factor scores were entered simultaneously as predictors). For RQ 2, the correlations among the latent factors describing the macro- and micro-level assessments of interpersonal functioning from the SEM were assessed. To facilitate interpretation of the figure, the correlations among latent factors and residual correlations were not included

Data were analyzed in several steps, presented schematically in Fig. 4.

#### Step 1. Computing the indices of the dynamic patterns of daily social experiences

For each daily diary item, three indicators were calculated for each participant: (i) a mean (M) score, describing the overall level, (ii) a standard deviation (SD) score, describing the variability, and (iii) a lag-1 autocorrelation (AR) score, describing the inertia in daily social experiences across the 6-month period. Since trends in the item scores might inflate the SD and AR values (Jahng et al., 2008), the linear trends were filtered out before computing these two indicators by regressing time on the scores of each item. The residuals resulting from this detrending procedure were used to compute the SD and AR. In order to estimate the AR, multilevel models were run for each item separately, using the *lme4* package in R, version 1.1–2.3 (Bates et al., 2015). In these models, the daily social experiences ratings of each participant on a given day *d* were predicted by the individual’s mean-centered social experience in the previous day *d-1;* random effects for the intercepts and slopes were estimated. The AR represents the regression coefficients (random slopes) of the social experience at *d-1* on the social experiences at *d*. These coefficients were extracted from each multilevel model and were aggregated into latent factors as described below (in Step 4).

#### Step 2. Extraction of factor scores describing affection and rejection separately at T1 and T3

Twelve confirmatory factor analyses (CFA) were fitted for each self- and other- reported measures of affection and rejection separately at T1 and T3, in order to explore the measurement models of the instruments used in the study. We calculated a weighted mean score by extracting the factor scores which were used in Step 3 of the analysis. Residuals were allowed to correlate for items that were highly similar in content (e.g.,”Does your mother/father blame you for everything?” and “If something happened at home, does your father/mother mainly blame you?”) or who belonged to the same subscale from the original questionnaire (e.g. the items from “anger outbursts” subscale from Parents Reaction self-report that were used as part of perceived parental rejection score). Table S1 from the Supplementary Material presents the model fit indices of the CFA models.

#### Step 3. Extraction of factor scores describing affection and rejection across adolescence

The 12 factor scores extracted in Step 2 were used in one CFA in which six latent factors were fitted, describing perceived parental affection and rejection, teacher and peer affection and peer rejection reported by parents and teachers across adolescence. Because each latent factor had two indicators (the factor scores extracted from the separate CFAs for each dimension at T1 and T3 of the TRAILS-CC study), the factor loadings were constrained to be equal in strength. Residuals were allowed to correlate between (i) the observed variables measured by the same instrument (e.g. T1 and T3 self-reports of peer affection measured using SPF questionnaire) and (ii) the variables referring to the relationship with the same persons at the same time point (e.g. self-reported peer affection and other- reported on peer rejection). Next, 6 latent factor scores were extracted using the ten Berge method (ten Berge et al., 1999). The ten Berge method was used in order to preserve the correlation among the latent factor scores from the CFA model (Logan et al., 2021).

#### Step 4. Exploring the research questions of the study

A structural equation model (SEM) was fitted to explore the longitudinal relationships between the quality of different relationships during adolescence and micro- and macro-level interpersonal functioning, as well as the cross-sectional relationships between macro- and micro-level assessments of interpersonal functioning. In this model, the factor scores extracted in Step 3 describing the quality of the relationships during adolescence were used as predictors (all factor scores simultaneously entered as predictors) for 7 latent factors describing the interpersonal functioning at the macro- and micro- level during young adulthood. For the macro-level, one latent factor was created including the mean score at baseline and post daily diary, with the factor loadings constrained to be equal in strength. The indices of daily social experiences (M, SD and AR scores) measured at micro-level computed in Step 1 were fitted into 6 latent factors, describing the mean level, variability, and inertia in the positive and negative daily social experiences (see Fig. 4). Residuals were allowed to be correlated among the indices (M, SD, AR) of the same item. A total of 7 regressions were run in the SEM. For the secondary research question, the correlations among the latent factors describing the interpersonal functioning at a macro- and micro-level during adulthood were investigated in the SEM.

Due to differences in variability among the variables, all scores were standardized before being entered in the CFA and SEM analyses. Model estimation was based on the maximum likelihood estimation with robust standard error (MLR) and missing data were handled using FIML in R using the package *lavaan* version 0.6–8 (Rosseel, 2012). Model fit was examined by the following criteria: the root mean square error of approximation (RMSEA), the comparative fit index (CFI) and the standardized root mean residual (SRMR). Model fit is considered acceptable if CFI > 0.90, RMSEA < 0.10 and SRMR < 0.09 (Hu & Bentler, 1999). Because of the exploratory nature of the study, the significance level of alpha was fixed at 0.05.

#### Sensitivity analysis- covariates inclusion

Based on previous studies suggesting that interpersonal functioning is associated with the sex (Rose & Rudolph, 2006), age, SES (Almquist & Brännström, 2014), and IQ (Murray et al., 2014), sensitivity analysis were performed to test if the effects remained significant after the inclusion for these covariates. For this purpose, the SEM presented in Step 4 was re-run including sex, age, parents’ SES, and IQ scores at T1 as predictors for the latent factors describing interpersonal functioning during adulthood alongside the factor scores describing the quality of social relationships during adulthood.

#### Post-hoc analysis

In the main analysis, we focused on the affection and rejection across adolescence, combining the measures at the ages 11.2 ± 0.4 and 16.0 ± 0.6 years old. However, from a developmental viewpoint the effects of parental, peer, and teacher affection and rejection on adult interpersonal functioning could be age dependent. To address this possibility, a separate SEM was run. In this SEM, the latent factors describing interpersonal functioning at macro- and micro- level during young adulthood were regressed on the 12 factors scores extracted in Step 2 describing interpersonal affection and rejection at T1 and T3 (all the factor scores were entered simultaneously in the analysis).

#### Missing data

The analytical sample was *n* = 122. For the interpersonal affection and rejection measured in the TRAILS-CC study there were two types of missing data in this sample: non-response at different questionnaires within each assessment (at T1 and T3) and non-participation at T3. At T1, the percentage of missing data due to non-response was: 3.28% for the peer and teacher affection and 9.02 % for teacher reported peer rejection. At T3, missing data due to non-participation at this assessment was 9.02%, resulting in a sample size of 111 youth at T3. In addition to the overall missingness due to non-participation in the study at T3, teacher-reported peer rejection contained an additional 22 persons with missing data (5.04%), resulting in a total sample of 89 for teachers reports of peer rejection at T3. Table S4 from the Supplementary materials presents the sample size for each of the measures at T1 and T3. Little’s MCAR test indicated that the data was missing completely at random *χ2* (106) = 114, *p* = 0.275, thus FIML was used in the SEM models to handle the missing data. Regarding the daily diary items, the M, SD, and AR were computed based on the responses from all the 122 participants who completed the daily diary study. Within this sample, on average, there were 22% of missing data due to non-response on given days. Data were not imputed to calculate the M, SD and AR. AR were estimated using multilevel models that perform very well with missing data (Bolger & Laurenceau, 2013). After computing each daily diary index (M, SD, AR), no missings were present in the data. No missing data were observed in the means scores of interpersonal functioning at baseline or post daily diary assessment.

## Results

### Descriptive results

Table 1 presents the descriptive statistics for demographic information and the interpersonal functioning measured at the micro- and macro-level during young adulthood. As factor scores were used in order to measure interpersonal affection and rejection during adolescence at T1 and T3, no descriptive statistics are presented. Rather, in order to be able to compare the sample characteristics with those used in previous studies, we present the descriptive statistics of the self- and other-reported parental, peer, and teacher affection and rejection scores at T1 and T3 in Table S4 in the Supplementary Materials. Table S5 in the Supplementary Materials shows the bivariate correlations among all variables used in the analysis of the present study.

**Table 1:** Demographics and descriptive statistics of the interpersonal functioning measured at the micro- (M, SD and AR for the daily diary items) and macro-level (at baseline and post daily diary assessment)

	N	M/%	SD	Min	Max
**Demographic information**					
Age T1	122	11.19	0.46	10.13	12.17
Age T3	111	15.96	0.62	14.66	17.40
Age T5	122	23.64	0.67	22.26	24.81
SES T1					
Low	122	20.5			
Medium	122	54.1			
High	122	25.4			
IQ T1	122	99.73	14.6	67	142
**Interpersonal functioning young adulthood**					
GVSG baseline	122	3.49	0.34	1.5	4
GVSG post	122	3.32	0.39	1.6	3.94
M social	122	57.82	14.58	7.14	98.83
M liked	122	58.14	15.34	3.06	98.37
M ease	122	58.97	14.68	3.82	93.79
M lonely	122	14.61	13.34	0.31	62.59
M annoyed	122	12.68	9.65	0.11	46.05
M fight	122	7.51	8.14	0.08	47.13
SD social	122	14.47	5.60	3.25	37.98
SD ease	122	14.67	5.71	3.52	42.46
SD liked	122	13.87	5.52	2.50	42.61
SD annoyed	122	9.36	5.20	0.40	28.99
SD lonely	122	10.83	7.01	0.46	31.86
SD fight	122	7.18	4.74	0.46	23.26
AR social	122	0.12	0.09	−0.10	0.48
AR ease	122	0.13	0.08	−0.09	0.55
AR liked	122	0.13	0.08	−0.09	0.47
AR annoyed	122	0.10	0.06	−0.09	0.30
AR lonely	122	0.16	0.10	−0.04	0.43
AR fight	122	0.09	0.07	−0.05	0.38

*M* mean, *SD* standard deviation, *AR* autocorrelations, *SES* socio-economic status, *GVSG* Groningen Social Behavior Questionnaire

*Daily diary items abbreviations*: social-‘I was social’; ease-‘I was at ease with others’; liked-‘I felt that others liked me’; annoyed-‘I felt that others were annoyed by me’; lonely-‘I felt lonely’; fight-‘I had a fight

### Step 1. Indices of daily social experiences

Regarding the daily social experiences during adulthood, on average, participants reported relatively high levels of daily positive social experiences (means = 58.97, 58.14, 57.82, item scales from 0 to 100) and relatively low mean of negative daily social experiences (means = 14.61, 12.68, 7.51, item scales from 0 to 100). Individuals’ responses varied across the 6 months of daily assessments, with mean within-person SD scores of 14.47, 14.67, 13.87 for the positive items and of 10.83, 7.18, 9.36 for the negative items. Regarding the autocorrelations at lag-1 (AR), the multilevel models suggested a significant spillover effect of social experiences from the previous day on the next day (*p* < 0.001) for each item. The mean AR values ranged from 0.10 (for the item “I felt that others were annoyed by me”) to 0.19 (for the item “I feel lonely”).

### Step 2. Extraction of factor scores describing affection and rejection separately at T1 and T3

Model fit indices for the 12 one-factor CFAs at T1 and T3 are presented in Table S1 in the Supplementary Materials. The model fit indices were acceptable (CFI ranged between 0.95–1, RMSEA between 0.000–0.07, SEMR between 0.001–0.05) and all factor loadings were significant (*p* < 0.01), with standardized values all >0.30.

### Step 3. Extraction of factor scores describing the quality of social relationships across adolescence

Model fit for the CFA fitting the factor scores extracted in Step 2 into six latent factors describing self- and other- reported affection and rejection across adolescence was acceptable (*χ2(df)* = 31.01 (25), CFI = 0.97, RMSEA = 0.04, SRMR = 0.06). Factor loadings for self-reported parental affection and rejection, peer and teacher affection and parent-reported peer rejection were all >0.30 and statistically significant (*p* < 0.001). The loadings for teacher-reported peer rejection were < 0.30 and non-significant (estimate values 0.26 and 0.24, *p* = 0.151). The estimates for teacher reported peer rejection are lower than the loadings for the other measures, probably because this is the only indicator for which the raters differed (i.e. different teachers) from T1 and T3. Figure 4 presents the standardized loadings for each latent factor fitted in the CFA in Step 3. The correlation between the factor scores show that self-reported parental, peers and teacher affection were moderately correlated (correlation coefficients ranging from 0.43 to 0.56), suggesting that adolescents who reported high affection from certain people (e.g. parents) are also more likely to report high affection from other people (e.g. teachers and peers). Similarly, self-reported parental rejection was moderately correlated with other-reported peer rejection (correlation coefficients ranging from 0.34 to 0.49) suggesting that youth experiencing parental rejection also experience higher peer rejection. Table S6 from the Supplementary material presents the correlation coefficients among the latent factors from the CFA.

### Step 4. Addressing the research questions of the study

Table 2 shows the coefficients of the final SEM model, in which the factor scores extracted from the CFA model in Step 3 were used to predict the latent factors describing interpersonal functioning in young adulthood at macro- and micro- level. Model fit indices suggested an acceptable fit to the data (*χ2 (df)* = 359.51(210), CFI = 0.91, RMSEA = 0.07, SRMR = 0.07). Factor loadings of the latent factors describing macro-level interpersonal functioning, mean levels, variability and inertia in positive and negative social experiences were > 0.30 and statistically significant (*p* < 0.01). Figure 4 presents the standardized loadings for each latent factor describing macro- and micro-level interpersonal functioning during adulthood from the SEM.

**Table 2:** Standardized regression coefficients derived from structural equation modeling (SEM) in which interpersonal functioning during young adulthood assessed at macro- and micro- level were predicted by self- and other-reported parental, peer and teacher affection and rejection during adolescence

	Parental affection		Parental rejection		Teacher affection		Classmate affection		Peer rejection- parent report		Peer rejection- teacher report								
	β (95% CI)	*p*	β (95% CI)	*p*	β (95% CI)	*p*	β (95% CI)	*p*	β (95% CI)	*p*	β (95% CI)	*p*	*1*.	*2*.	*3*.	*4*.	*5*.	*6*.	*7*.
1.Interpersonal functioning (GVSG)	0.06 (−0.19 to 0.33)	0.588	0.15 (−0.23 to 0.56)	0.416	−0.07 (−0.52 to 0.36)	0.724	0.25 (−0.04 to 0.60)	0.096	−0.07 (−0.36 to 0.20)	0.570	−0.28 (−0.54 to −0.06)	*0.01*	–						
2.Mean positive daily social experiences	0.08 (−0.11 to 0.29)	0.412	0.20 (−0.17 to 0.58)	0.292	0.02 (−0.35 to 0.40)	0.893	0.03 (−0.25 to 0.31)	0.828	−0.06 (−0.28 to 0.15)	0.566	−0.15 (−0.34 to 0.02)	0.08	***0.57***	–					
3.Mean negative daily social experiences	−0.08 (−0.33 to 0.14)	0.449	−0.27 (−0.65 to 0.04)	0.09	0.004 (−0.33 to 0.34)	0.981	−0.12 (−0.43 to 0.16)	0.378	0.008 (−0.27 to 0.29)	0.952	0.42 (0.19 to 0.74)	**0.001**	−0.11	−0.12	–				
4.Variability positive daily social experiences	0.04 (−0.19 to 0.28)	0.701	−0.04 (−0.52 to 0.42)	0.841	−0.13 (−0.61 to 0.34)	0.579	0.01 (−0.51 to 0.53)	0.969	0.01 (−0.40 to 0.44)	0.934	0.06 (−0.15 to 0.28)	0.555	−0.09	−0.14	−0.17	–			
5.Variability negative daily social experiences	−0.06 (−0.36 to 0.22)	0.660	−0.37 (−0.86 to 0.06)	0.09	−0.29 (−0.76 to 0.14)	0.177	−0.03 (−0.55 to 0.48)	0.887	0.09 (−0.31 to 0.52)	0.619	0.30 (0.06 to 0.60)	*0.01*	**−0.35**	**−0.22**	0.31	***0.58***	–		
6.Inertia positive daily social experiences	−0.07 (−0.35 to 0.21)	0.616	−0.24 (−0.67 to 0.17)	0.254	−0.16 (−0.53 to 0.20)	0.387	0.13 (−0.28 to 0.54)	0.534	0.16 (−0.20 to 0.53)	0.388	0.09 (−0.18 to 0.36)	0.520	**−0.36**	*−0.21*	−0.16	0.03	−0.12	–	
7.Inertia negative daily social experiences	0.08 (−0.24 to 0.43)	0.575	−0.29 (−0.97 to 0.30)	0.309	−0.22 (−0.76 to 0.27)	0.361	−0.05 (−0.67 to 0.55)	0.848	−0.35 (−0.98 to 0.20)	0.202	0.26 (−0.14 to 0.72)	0.184	−0.20	0.05	*0.42*	−*0.42*	0.01	0.46	–

Significant effects are marked in bold and italics for ***p*** < ***0.001****,* in bold for ***p*** < **0.01**, in italics for *p* < *0.05*. SEM are based on maximum likelihood with robust standard error estimation (MLR), sample size n = 122

In the right part of the table, correlation coefficients among the latent factors describing macro- and micro- level interpersonal functioning during young adulthood are presented

The SEM results indicated several statistically significant (*p* < 0.05) longitudinal associations: higher peer rejection reported by teachers during adolescence predicted lower levels of interpersonal functioning at the macro-level, higher mean levels of negative daily social experiences, and higher variability in negative daily social experiences during young adulthood. Parental, peer and teacher acceptance and rejection during adolescence explained between the 1 to 18% of variance in the latent factors describing interpersonal functioning during adulthood (13% for interpersonal functioning at macro-level, 3% for the mean level of positive daily social experiences, 15% for the mean level of negative daily social experiences, 1% for the variability in positive daily social experiences, 11% for the variability in negative social experiences, 2% in the inertia in positive daily social experiences, 18% in the inertia in the daily negative social experiences).

Addressing the secondary research question, the correlations among the latent factors from the SEM showed that macro-level interpersonal functioning during young adulthood was positively correlated with the mean level of positive daily social experiences and negatively correlated with the variability in daily negative social experiences and inertia in daily positive social experiences. Table 2 presents the correlations between the latent factors from the SEM model describing macro- and micro-level interpersonal functioning.

### Sensitivity analysis results

After the inclusion of all covariates (sex, IQ, SES of the parents, age of the participants at T1) in the SEM model run in Step 4, the effects remained similar in strength and significance. Table S7 from the Supplementary Materials shows the results of the SEM after the inclusion of covariates.

### Post-hoc analysis results

The results of the SEM in which latent factors capturing macro- and micro-level interpersonal functioning during adulthood were regressed on parental, peer and teacher affection and rejection at T1 and T3 confirmed the results of the SEM run in the Step 4. More precisely, the results indicated that peer rejection reported by teachers at both T1 (β = 0.45, *p* < 0.001) and T3 (β = 0.29, *p* = 0.026) predicted higher mean levels of daily negative social experiences. Furthermore, higher peer rejection reported by teachers at T3 predicted lower levels of interpersonal functioning assessed at macro-level (β = −0.30, *p* = 0.026) and higher variability in the negative daily social experiences (β = 0.24, *p* = 0.018). Additional results were observed: higher self-reported peer affection at T3 predicted higher macro-level interpersonal functioning levels (β = 0.30, *p* = 0.028) and higher peer affection at T1 predicted higher variability in positive daily social experiences (β = 0.25, *p* = 0.048). Higher self-reported teacher affection at T3 predicted lower variability in daily negative social experiences (β = −0.32, *p* = 0.01) and higher self-reported parental affection at T3 predicted lower mean levels of daily negative social experiences (β = −0.21, *p* = 0.043). Table S8 from the Supplementary material presents the results of the post-hoc analysis. All the analysis performed, all data exclusions, and all the variables that were included in the final statistical analysis were reported.

## Discussion

Adolescence represents an important period for social and self-development, when social relationships are increasingly important and rewarding (Kilford et al., 2016). The quality of social relationships during adolescence is believed to impact adult interpersonal functioning, but few studies focused on the joint impact of parental, peer, and teacher affection and rejection on the daily, micro-level social experiences as well as general, macro-level interpersonal functioning in young adulthood. Furthermore, little is known about the specific associations between macro-level interpersonal functioning and dynamic patterns of daily social experiences. The present study aimed to investigate the simultaneous effects of parental, peers, and teacher affection and rejection during adolescence and interpersonal functioning measured at macro- (i.e. across several months) and micro-level (i.e. daily social experiences) during young adulthood, using a multi-informant, prospective longitudinal study. The results suggested that higher levels of teacher-reported peer rejection during adolescence were associated with lower levels of interpersonal functioning assessed at the macro-level and higher mean levels and higher variability in daily negative social experiences during young adulthood. The cross-sectional findings showed that, in young adulthood, higher levels of macro-level interpersonal functioning were associated with higher mean levels and lower inertia in positive daily social experiences, as well as with lower variability in negative daily social experiences. The results of the present study add evidence to the existing literature by showing that the peer rejection during adolescence impacts not only interpersonal functioning assessed at the macro-level, but also specific daily patterns of negative social experiences.

At the macro-level, the results of the main analysis suggest that peer rejection reported by teachers during adolescence predicted lower interpersonal functioning in young adulthood. This finding is in line with previous studies showing that the quality of adolescents’ relationships with peers are more salient and play a more central role in the future interpersonal development than the other relationships (i.e. with parents or teachers) (Kilford et al., 2016). Interestingly, only teacher-reported peer rejection was associated with daily social experiences. The role of teachers in identifying emotional and behavioral problems has been previously recognized (e.g. Navarro et al., 2020). Adolescents spend more time with peers in school related activities (Junge et al., 2020) and some aspects of social interactions might manifest predominantly in school settings. Thus, teachers might observe youth in specific social contexts to which parents do not have access. This finding stresses the importance of using multi-informant assessments of social relationships during adolescence. However, the results should be interpreted with caution, since the loadings for the latent factor describing teacher-report peer rejection across adolescence were somewhat low, probably due to different teachers at T1 and T3. This, in contrast to parent and self-report who remain the same over time. Nevertheless, the post-hoc analysis performed with the separate scores at T1 and T3 as predictors for interpersonal functioning during adulthood indicate that peer-rejection reported by teachers at T3 predicted macro-level interpersonal functioning. Furthermore, the results of the post-hoc analysis also reveal that self-reported peer affection at T3 predicted higher interpersonal functioning during young adulthood, which might indicate that both positive (affection) and negative (rejection) aspects of adolescents’ relationship with peers impact future interpersonal functioning. These results may also suggest that the developmental period (i.e. mid-adolescence) at which peer affection and rejection were experienced matters. However, replication of these post-hoc findings is necessary.

In addition to the associations with the macro-level interpersonal functioning, peer rejection reported by teachers also predicted higher mean levels and higher variability in negative daily social experiences during young adulthood. The present study is the first to focus on associations between affection and rejection during adolescence and daily social experiences during young adulthood, and thus, we cannot directly compare the current findings to previous work. Relationships with peers, compared with those with adults, are less hierarchical, thus they might facilitate the learning of social skills that are necessary for adult daily social interactions (Miljkovitch et al., 2021). Peer rejection during adolescence might hinder the development of social skills and socioemotional processes (Junge et al., 2020), leading to increased difficulties in everyday social interactions later in life. The associations between peer rejection during adolescence and high variability in negative daily social experiences could add additional insight into the mechanisms through which peer rejection during adolescence impacts lower interpersonal functioning during adulthood. High variability in daily social experiences has previously been linked to unstable representations about others (Campbell et al., 2010) and to difficulties in emotion regulation (e.g. Thompson et al., 2017), which, in turn, have been previously associated with peer rejection during adolescence (e.g. Herd & Kim-Spoon, 2021). Furthermore, peer rejection has been previously associated with hypersensitivity to negative social experiences (e.g. Will et al., 2016), which might be reflected in the increased variability in daily negative social experiences. Post-hoc analysis results confirm that higher teacher reports of peer rejection at both T1 and T3 predict higher mean levels of negative social experiences. Furthermore, post-hoc analysis showed that higher self-reported parental affection at T3 predicted lower mean levels of daily negative social experiences and that higher teacher affection during at T3 predicted lower variability in daily negative social experiences. These results might highlight the importance of the role of interpersonal affection in broader interpersonal contexts for the development of stable and controlled reactions to everyday social contexts during young adulthood.

The cross-sectional results are in line with previous studies that showed associations between macro-levels of interpersonal functioning and mean level of daily social experiences (e.g. Schneider et al., 2017). The present study adds to the existing literature by highlighting that interpersonal functioning is related not only with mean levels, but also with specific aspects of dynamic patterns of daily positive and negative social experiences. The results suggest that interpersonal functioning is characterized by flexible (low inertia), yet relatively stable (low variability) daily social experiences, which echoes with theoretical frameworks on interpersonal processes (Pincus & Wright, 2011). Good interpersonal functioning might impact the dynamics of daily social experiences, buffering the effect of daily stressors (Lippold et al., 2016), leading to more stable daily social experiences. Relationships that are perceived as less warm and supportive might represent a stressor in themselves, leading to uncertainty and hypersensitivity to social stimuli, reflected in greater variability and inertia of daily social experiences (Janssen et al., 2021). On the other hand, dynamic patterns of daily (social) experiences have been hypothesized to represent early indicators of interpersonal dysregulations (Wichers, 2014) that could predict future interpersonal problems (Schreuder et al., 2020). Future studies repeatedly assessing interpersonal functioning at the macro- and micro-levels are needed in order to further disentangle the relations between different time-scales of interpersonal processes, which could shed light into the mechanisms through which daily social experiences are ingrained into macro-level constructs.

### Strengths and limitations

The main strength of this study lies in its design: a prospective longitudinal design complemented with 6 months of daily diary assessments during adulthood, spanning in total almost 12 years and linking micro-level with macro-level assessments. Furthermore, the present study focused on the quality of different relationships by using a multi-informant approach at two different time points across early and mid-adolescence. Additionally, the daily diary study spanned 6 months, a much longer period than typically used in research focusing on interpersonal functioning using ILD.

There are also several limitations that have to be acknowledged. First, the sample size is relatively small (i.e. for investigating the macro-level relations) and the micro-level results should be replicated in independent samples. Regarding the quality of the relationships during adolescence, one limitation of the study is that multi-informant reports were not used for the assessment of the quality of all relationships. For instance, adolescents spend a substantial amount of time with their friends in the absence of adults, thus peer-reports could shed light into aspects of social relationships that were not covered by the measures used in the present study. Another limitation is that consistency over time in teachers’ ratings of peer rejection was low (as indicated by low loading). Nevertheless, the post-hoc analysis using teacher-reports of peer rejection separately from T1 and T3 as predictors for interpersonal functioning during adulthood confirm the results of the main SEM, which suggests that although teachers reports at T1 and T3 might not be highly correlated, both of them are informative for future interpersonal functioning. Furthermore, the objective aspects of interpersonal functioning (e.g. number of friends or number of social interactions per day) were not measured at either a macro- or micro-level during adulthood. However, this choice was purposely made as previous studies suggested that the quality of social relationships assessed at a macro-level is associated to a greater extent with subjective rather than with objective measures of daily interpersonal functioning (e.g. Achterhof et al., 2022). At the same time, however, some studies indicate that dynamic patterns in objective aspects of interpersonal functioning (e.g. inertia in the amount of time being alone) are predictive for mental health problems (Elmer et al., 2020). Future studies ideally should combine subjective and objective aspects of daily social interactions in order to better grasp the complexity of interpersonal functioning. Although the interpretations of variability and inertia as extreme responses to social contexts make good sense, the contexts in which social experiences emerged were not directly investigated in the present study. Previous research suggests that social experiences vary depending on contextual factors, for example the type of social interaction (e.g. Hur et al., 2019) and the presence of stressful events (e.g. Sitko et al., 2016). Nevertheless, it has been suggested that dynamic patterns in (social) experiences reflect internal processes (e.g. rigidity) of responding to (social) environments (Houben et al., 2015). By combining ILD with other methodologies (e.g. experimental, observational designs) that provide information about contextual and individual (socio-cognitive) factors, future studies could shed light on the factors underpinning increased variability and inertia in daily social experiences. Finally, given the nature of the sample used in the present study, which was composed of youth at-risk of presenting mental health problems, the present results cannot be extrapolated to other populations.

## Conclusion

The quality of relationships during adolescence has been associated with interpersonal functioning during adulthood. However, previous studies focused on affection and rejection in specific contexts (e.g. only with parents or peers) and assessed interpersonal functioning only at the macro-level (i.e. across several months or years). The present study contributed to prior research by simultaneously investigating the impact of self- and other- reported parental, peer, and teacher affection and rejection during adolescence on the micro-level (daily) social experiences as well as on the macro-level interpersonal functioning in young adulthood. Furthermore, this study aimed to understand how aspects of dynamic patterns in daily social experiences were associated with macro-level interpersonal functioning. The results showed that higher peer rejection reported by teachers during adolescence was associated with lower interpersonal functioning assessed at the macro-level, as well as with higher mean levels and higher variability in daily negative social experiences in young adulthood. These results highlight the long-term effects of peer rejection during adolescence, not only on the general, macro-level interpersonal functioning, but also on everyday social experiences. The present study also suggests that the effects of peer rejection might impact unstable self and other representations and emotion dysregulation in social contexts, as reflected in higher variability in negative daily experience. The cross-sectional results suggested that, in young adulthood, better interpersonal functioning was correlated with higher mean levels and lower inertia in daily positive social experiences and lower variability in negative daily social experiences. The results also showed that macro-level assessments of interpersonal functioning were associated not only with mean levels, but also with specific dynamic patterns of daily social experiences. Future studies are necessary for understanding the specific mechanisms through which the quality of social relationships during adolescence and young adulthood impact daily dynamics in social experiences, integrating both macro- and micro-levels of investigation.

## Supplementary information

Supplementary Information
