# Supplementary material for: The Associations of Affection and Rejection During Adolescence with Interpersonal Functioning in Young Adulthood: A Macro- and Micro- Level Investigation Using the TRAILS TRANS-ID Study
Source: J Youth Adolesc. 2022 Jul 19;51(11):2130–45. doi: 10.1007/s10964-022-01660-y (PMC9508212; doi:10.1007/s10964-022-01660-y)
Supplement: Supplementary file 1 — Supplementary Information [file 10964_2022_1660_MOESM1_ESM.docx]

The Associations of Affection and Rejection during Adolescence with Interpersonal Functioning in Young Adulthood:

a Macro- and Micro- Level Investigation using the TRAILS TRANS-ID Study

*Larisa Morosan, Johanna T. W. Wigman, Robin N. Groen,* *Marieke J. Schreuder, Marieke Wichers, Catharina A. Hartman*

*University of Groningen, University Medical Center Groningen, ICPE, The Netherlands*

**Contents**

[Measures 2](#_Toc102482955)

[Table S1. Model fit indices from the 12 separate one-factor CFA models 3](#_Toc102482956)

[Table S2. The items measuring the quality of social relationships during adolescence at T1 and T3 in the TRAILS-CC study. 3](#_Toc102482957)

[Table S3. Items measuring the interpersonal functioning at a macro-level using the GVSG in the TRAILS-TRANS-ID study. 5](#_Toc102482958)

[Table S4. Descriptive statistics for the variables measured at T1 and T3 in the TRAILS study. 6](#_Toc102482959)

[Correlation table 7](#_Toc102482960)

[Table S5. Bivariate correlations for the variables included in the SEM model. 7](#_Toc102482961)

[Overview of the final model including factor loadings 9](#_Toc102482962)

[Figures S1. Overview of the steps taken for the statistical analysis, including the loadings for each latent factor fitted in Step 2 and Step 3. 9](#_Toc102482963)

[**Table S6.** Correlations among latent factors describing interpersonal affection and rejection during adolescence, from the CFA model. 9](#_Toc102482964)

[Sensitivity analysis results 10](#_Toc102482965)

[Table S7. Standardized regression coefficients derived from structural equation modeling(SEM)10](#_Toc102482966)

[Post hoc analysis results 12](#_Toc102482967)

[Table S8. Standardized regression coefficients derived from structural equation modeling(SEM)12](#_Toc102482968)

[References 14](#_Toc102482970)

# Measures

**Self-reported parental affection.** At T1, parental affection was assessed using a set of 7 items from “emotional warmth” (all the items are presented in Tables S2) subscale of the Egna Minnen Beträffande Uppfostran (My Memories of Upbringing) for Children (EMBU-C) (Markus et al., 2003). The items referring to each parent were highly correlated (r=0.51- 0.74), thus they were averaged. At T3, parental affection was assessed using 7 items describing problem-solving behaviors and parental solicitations from the parental reactions to child behaviors (based Tilton-Weaver et al., 2010). The items were selected based on their face validity, in order to contain emotional aspects of relations with parents (i.e. emotional warmth) and to match to a certain extent the items from the questionnaire investigating teachers’ and peers’ affection. Four CFAs were conducted to test the model fit of the perceived parental affection and rejection subscales, at each time point separately, using the items selected (mean scores for mother and father). All factor loadings were significant (*p*<0.001) with standardized values above 0.30 (ranging from 0.49 and 0.76). Model fit indices are presented in Table S1.

**Self-reported parental rejection.** At T1, parental rejection was assessed using 5 items (all the items are presented in Tables S2) from the “parental rejection and overprotection” subscales of the Egna Minnen Beträffande Uppfostran (My Memories of Upbringing) for Children (EMBU-C) (Markus et al., 2003). At T3, parental rejection was assessed using 5 items describing parental guilt inducing and angry outbursts behaviors (based on Tilton-Weaver et al., 2010). The items referring to each parent were highly correlated (*r*=0.46- 0.75), therefore were averaged. The items were selected based on their face validity, in order to contain emotional aspects of relations with parents (e.g. rejection, blaming, guilt inducing behaviors), without focusing on punishment or preoccupation with school performances (aspects that were assessed Parental reaction questions and were not included in the present study). Four CFAs were conducted to test the model fit for the perceived parental affection and rejection subscales, at each time point separately, using the items selected (mean scores for mother and father) All factor loadings were significant (*p*<0.001) with standardized values above 0.30 (ranging from 0.36 and 0.86). Model fit values are presented in Table S1.

**Self-reported teacher and peer affection.** Perceived teachers’ and classmates’ affection was assessed at T1 and T3 with the items of the affection and behavioral confirmation subscales of the Social Production Function Questionnaire (SPF; Ormel et al., 1997) (see Table S2, for the specific items included). Four CFAs were conducted to assess the fit of the measurement model for perceived teacher and peer affection at each time point. One item from the teacher affection subscale (“When someone else does something in class, the teachers blame me”) had low loadings (-0.11) for the T1 assessment, thus it was excluded from the analysis for both time points. All factor loadings were significant (*p*<0.001), with standardized values above 0.30 (ranging from 0.38 and 0.79). Model fit values are presented in Table S1.

**Parents and teacher reported peer rejection.** Peer rejection was reported by parents and teachers and T1 and T3 using the Child Behavior Checklist (CBCL) and Teacher’s Report Form (TRF) (Achenbach, 2001). Four items (see Tables S2) were selected from the social problems subscale of these questionnaires to match to a certain extent the self-reported measures. Four CFAs were conducted to assess the fit of the measurement models at each time point separately. All loadings were significant (*p*<0.01), with standardized values above 0.30 (ranging between 0.49-0.89). Model fit values are presented in Table S1.

The factor scores resulting from the 12 CFAs testing the measurement models for each domain separately at T1 and T3 were extracted as indicators of weighted sum scores. The extracted factor scores were highly correlated with the mean scores of the items selected for each domain (*r*=0.81 - 0.99).

**Table S1**. Model fit indices from the 12 one-factor CFA models conducted to explore the model fit of the subscales used to measure interpersonal affection and rejection at T1 and T3 in the TRAILS-CC study. Model estimation was based on maximum likelihood estimation with robust standard errors (MLR)**.** All loadings were higher than 0.30 and significantly different from zero (*p*<0.01).

|  | **N** | **χ2 (df)** | **CFI** | **RMSEA** | **SRMR** |
| --- | --- | --- | --- | --- | --- |
| Parental affection T1 | 122 | 19.30 (13) | 0.97 | 0.06 | 0.04 |
| Parental affection T3 | 111 | 20.59 (14) | 0.95 | 0.07 | 0.05 |
| Parental rejection T1 | 122 | 5.55 (4) | 0.98 | 0.05 | 0.02 |
| Parental rejection T3 | 111 | 1.19 (4) | 1 | 0.000 | 0.01 |
| Teacher affection T1 | 118 | 42.03(20) | 0.95 | 0.07 | 0.05 |
| Teacher affection T3 | 111 | 24.36 (19) | 0.98 | 0.05 | 0.04 |
| Peer affection T1 | 118 | 29.10 (19) | 0.97 | 0.07 | 0.03 |
| Peer affection T3 | 111 | 25.02 (19) | 0.98 | 0.05 | 0.03 |
| Peer rejection parents reported T1 | 122 | 0.005 (1) | 1 | 0.000 | 0.003 |
| Peer rejection parents report T3 | 111 | 0.004 (1) | 1 | 0.000 | 0.001 |
| Peer rejection teacher report T1 | 111 | 0.20(1) | 1 | 0.000 | 0.003 |
| Peer rejection teacher report T3 | 89 | 0.003(1) | 1 | 0.000 | 0.001 |

## **Table S2.** The items measuring the quality of social relationships during adolescence at T1 and T3 in the TRAILS-CC study.

| **Construct and instrument for each assessment time point** | **Items** | **Item scale** | **Cronbach’s alpha of the selected items** |
| --- | --- | --- | --- |
| ***Self-reports*** | | | |
| **Parental affection** | |  |  |
| T1  (EMBU-C) | Do you feel that your father / mother loves you? | 1 (never) to  4 (almost always) | 0.80^a^ |
|  | Does your father / mother ever hug you? |  |  |
|  | When things are going badly for you, is your father / mother trying to comfort or help you? |  |  |
|  | Do you have the feeling that your father / mother is willing to help you when you have to do something difficult? |  |  |
|  | Does your father / mother clearly show that he / she loves you? |  |  |
|  | Do you think your father / mother takes into account what you think of something? |  |  |
|  | Do you have the feeling that your father / mother likes to be with you? |  |  |
| T3  (Parental reactions) | Your mother/father is clear about what she thinks, but is also open to discussion. | 0 (never) to  4 (almost always) | 0.79 ^a^ |
|  | Your mother/father really wants to understand why you did what you did. |  |  |
|  | Your mother/father tries to understand how you thought and felt. |  |  |
|  | Your mother/father tries to discuss it with you without creating new conflicts. |  |  |
|  | Does your mother/father start a conversation with you to talk about things you have been through? |  |  |
|  | Did your mother/father start a conversation with you about your free time in the past month? |  |  |
|  | Does your mother/father start a conversation with you about things that happened during a normal school day? |  |  |
| **Parental rejection** | |  |  |
| T1  (EMBU-C) | Does your father / mother blame you for everything? | 1 (never)  4 (almost always) | 0.68 ^a^ |
|  | If something happened at home, does your father / mother mainly blame you? |  |  |
|  | Does your father / mother ever act harshly and unkindly towards you? |  |  |
|  | Does your father / mother ever act in such a way that you feel small? |  |  |
|  | Is your father / mother ever angry or sad with you without saying why? |  |  |
| T3  (Parental reactions) | Your mother/father is avoiding you | 0 (never) to  4 (almost always) | 0.73 ^a^ |
|  | Your mother/father is quiet and cold to you |  |  |
|  | Your mother/father will not talk to you for a long time |  |  |
|  | Your mother/father has outbursts of anger and you get turned on your head |  |  |
|  | Your mother/father argues with you and complains loudly |  |  |
| **Teachers affection** | | 1 (never) to  5 (always) | 0.85 /0.85 ^b^ |
| T1 & T3  (SPF) | Most teachers help me if there is anything. |  |  |
|  | Most teachers take my feelings into account. |  |  |
|  | Most teachers are satisfied with me as I am. |  |  |
|  | I can really trust most teachers. |  |  |
|  | Most teachers like it when I help them. |  |  |
|  | Most teachers think I can help others well. |  |  |
|  | Most teachers think I behave well. |  |  |
|  | Most teachers like me. |  |  |
| **Classmates affection** | | 1 (never) to  5 (always) | 0.90/ 0.90 ^b^ |
| T1& T3  (SPF) | Most classmates like to do things with me. |  |  |
|  | Most of my classmates help me if there is anything. |  |  |
|  | Most classmates take my feelings into account. |  |  |
|  | Most classmates enjoy being with me. |  |  |
|  | Most of my classmates think that I am well behaved. |  |  |
|  | Most classmates like it when I help them. |  |  |
|  | I can really trust most of my classmates. |  |  |
|  | Most of my classmates are satisfied with me as I am. |  |  |
| ***Other reports*** | | | |
| **Peers rejection -parents and teachers reports** | |  |  |
| T1 & T3  (CBCL & TRF) | Others boy/girls do not like him/her | 0 (Not at all) to  3 (Often) | TRF: 0.86/0.78 ^b^  CBCL: 0.78/0.77 ^b^ |
|  | Does not get along well with other boys / girls  Gets teased a lot |  |  |
|  | Has the feeling that others are after him/her |  |  |
|  |  |  |  |

^a^ Cronbach’s alpha values for the mean items scores

^b^ Cronbach’s alpha for items referring to T1 and T3, respectively

## **Table S3**. Items measuring the interpersonal functioning at a macro-level using the GVSG in the TRAILS-TRANS-ID study.

| **Items** | **Item scale** | **Cronbach’s alpha of the selected items** |
| --- | --- | --- |
| I got along well with my parents. | 1 (never) to  4 (always) | 0.65/ 0.74 * |
| I could talk with (one of) my parents about my personal problems. |  |  |
| If necessary, I could visit my parents for help and support. |  |  |
| My parents could come to me if they need help or support. |  |  |
| I've been avoiding my parents lately. |  |  |
|  |  |  |
| I got on well with my friends and acquaintances. |  |  |
| I could talk to at least one friend about my problems or the things that really bother me. |  |  |
| I have spoken by phone or personally with my friends and / or close acquaintances. |  |  |
| I enjoyed spending time with my friends and / or good acquaintances. |  |  |
| My friends and / or close acquaintances and I have done things together. |  |  |
|  |  |  |
| I got along well with my partner. |  |  |
| I could manage my personal problems with my partner by talking. |  |  |
| My partner and I are a good sexual match. |  |  |
| My partner has made himself/herself clear lately to be annoyed by my behavior. |  |  |
| I've been avoiding my partner lately. |  |  |
|  |  |  |
| I got on well with my fellow students /fellow students. |  |  |
|  |  |  |
| I got on well with others in my work(colleagues, suppliers, customers, etc.) |  |  |

*Cronbach’s alpha for all the items included in the mean score of interpersonal functioning at baseline and at post daily diary assessment

**Table S4.** Descriptive statistics for the mean scores of parental, teacher and peer affection and rejection measured at T1 and T3 in the TRAILS study. These variables were not used in the analysis, but they are presented for descriptive reasons. Factor scores extracted from the 12 CFAs were used in the main analysis of the study; the factor scores were highly correlated with the mean scores presented below, as described above (pages 2-3, Table S2).

|  | **N** | **M/ %** | **SD** | **Min** | **Max** | **Informant** |
| --- | --- | --- | --- | --- | --- | --- |
| Parental affection T1 | 122 | 3.16 | 0.56 | 1.19 | 4.00 | P |
| Parental affection T3 | 111 | 2.16 | 0.70 | 0.00 | 3.86 | P |
| Parental rejection T1 | 122 | 1.54 | 0.37 | 1.00 | 2.60 | P |
| Parental rejection T3 | 111 | 0.54 | 0.47 | 0.00 | 2.40 | P |
| Teacher affection T1 | 118 | 3.98 | 0.68 | 1.00 | 5.00 | P |
| Teacher affection T3 | 111 | 3.67 | 0.63 | 1.75 | 4.88 | P |
| Peer affection T1 | 118 | 3.39 | 0.82 | 1.13 | 5.00 | P |
| Peer affection T3 | 111 | 3.52 | 0.75 | 1.00 | 5.00 | P |
| Peer rejection CBCL T1 | 122 | 0.70 | 0.48 | 0.00 | 2.00 | Pa |
| Peer rejection CBCL T3 | 111 | 0.36 | 0.41 | 0.00 | 2.00 | Pa |
| Peer rejection TRF T1 | 111 | 0.47 | 0.55 | 0.00 | 1.75 | T |
| Peer rejection TRF T3 | 89 | 0.32 | 0.42 | 0.00 | 2.00 | T |

CBCL-Child Behavior Checklist, TRF-Teacher’s Report Form, P-Participant, Pa-Parent, T-Teacher

# Correlation table

**Table S5**. Bivariate correlations for the variables included in the SEM model. Correlations are computed on the raw data (n=83-122).

|  | 1 | 2 | 3 | 4 | 5 | 6 | 7 | 8 | 9 | 10 | 11 | 12 | 13 | 14 | 15 | 16 | 17 |
| --- | --- | --- | --- | --- | --- | --- | --- | --- | --- | --- | --- | --- | --- | --- | --- | --- | --- |
| 1. Parental affection T1 | — |  |  |  |  |  |  |  |  |  |  |  |  |  |  |  |  |
| 2. Parental affection T3 | **0.22** | — |  |  |  |  |  |  |  |  |  |  |  |  |  |  |  |
| 3. Parental rejection T1 | **-0.21** | *-0.21* | — |  |  |  |  |  |  |  |  |  |  |  |  |  |  |
| 4. Parents’ rejection T3 | 0.06 | -0.03 | 0.16 | — |  |  |  |  |  |  |  |  |  |  |  |  |  |
| 5. Teacher affection T1 | ***0.34*** | 0.01 | -0.14 | -0.07 | — |  |  |  |  |  |  |  |  |  |  |  |  |
| 6. Teacher affection T3 | 0.15 | *0.20* | *-0.22* | *-0.19* | **0.26** | — |  |  |  |  |  |  |  |  |  |  |  |
| 7. Peer affection T1 | 0.17 | -0.02 | -0.06 | **-0.26** | ***0.54*** | 0.12 | — |  |  |  |  |  |  |  |  |  |  |
| 8.Peer affection T3 | 0.18 | 0.03 | -0.12 | 0.09 | *0.24* | *0.21* | ***0.32*** | — |  |  |  |  |  |  |  |  |  |
| 9. Peer rejection-CBCL T1 | 0.13 | 0.05 | -0.004 | **-0.26** | -0.04 | 0.03 | ***-0.31*** | *-0.21* | — |  |  |  |  |  |  |  |  |
| 10. Peer rejection-CBCL T3 | -0.12 | 0.08 | -0.009 | 0.09 | *-0.21* | -0.08 | *-0.22* | ***-0.45*** | ***0.45*** | — |  |  |  |  |  |  |  |
| 11. Peer rejection- TRF T1 | -0.01 | 0.09 | -0.08 | *0.23* | -0.13 | *-*0.01 | ***-0.34*** | **-0.30** | ***0.43*** | **0.26** | — |  |  |  |  |  |  |
| 12. Peer rejection-TRF T3 | -0.06 | -0.05 | 0.12 | 0.03 | -0.07 | 0.14 | 0.01 | **-0.32** | 0.07 | ***0.45*** | 0.06 | — |  |  |  |  |  |
| 13. GVSG baseline | <0.001 | 0.07 | 0.004 | 0.10 | 0.03 | -0.01 | 0.001 | **0.33** | -0.05 | **-0.21** | -0.007 | **-0.33** | — |  |  |  |  |
| 14. GVSG post | 0.17 | -0.01 | -0.06 | 0.008 | 0.01 | -0.05 | 0.14 | *0.21* | -0.17 | *-0.17* | -0.16 | -*0.25* | ***0.55*** | — |  |  |  |
| 15. M social | **0.22** | 0.13 | <0.001 | **0.25** | 0.03 | 0.12 | -0.02 | ***0.35*** | 0.05 | -0.09 | -0.07 | -0.08 | ***0.40*** | ***0.43*** | — |  |  |
| 16. M liked | 0.15 | 0.05 | -0.03 | *0.23* | -0.12 | 0.11 | -0.14 | **0.27** | 0.09 | -0.07 | -0.10 | -0.11 | ***0.35*** | ***0.45*** | ***0.86*** | — |  |
| 17. M ease | 0.14 | 0.05 | -0.03 | *0.22* | -0.06 | 0.15 | -0.07 | ***0.33*** | 0.02 | -0.12 | -0.10 | -0.09 | ***0.38*** | ***0.46*** | ***0.90*** | ***0.94*** | — |
| 18. M lonely | -0.04 | 0.06 | -0.03 | -0.03 | 0.10 | 0.13 | 0.01 | **-0.29** | 0.04 | 0.14 | *0.22* | **0.28** | ***-0.40*** | ***-0.41*** | ***-0.31*** | ***-0.34*** | ***-0.36*** |
| 19. M annoyed | -0.09 | -0.06 | -0.01 | -0.02 | -0.05 | 0.03 | -0.11 | *-0.23* | 0.09 | 0.13 | **0.30** | **0.24** | -0.13 | -0.15 | -0.09 | -0.13 | -0.15 |
| 20. M fight | -0.03 | -0.14 | -0.03 | 0.01 | -0.08 | 0.05 | -0.10 | -0.17 | 0.005 | 014 | *0.24* | *0.25* | -0.06 | -0.03 | -0.05 | -0.08 | -0.09 |
| 21. SD social | -0.15 | 0.06 | 0.09 | 0.07 | -0.07 | *-0.23* | -0.03 | -0.17 | 0.13 | 0.11 | 0.09 | -0.03 | -0.08 | -0.15 | *-0.23* | *-0.18* | **-0.24** |
| 22. SD ease | 0.01 | 0.05 | -0.03 | 0.03 | 0.05 | -0.16 | 0.03 | -0.12 | 0.09 | 0.02 | 0.07 | -0.05 | -0.08 | -0.02 | -0.03 | -0.09 | -0.17 |
| 23. SD liked | 0.02 | <0.001 | -0.03 | -0.002 | 0.13 | -0.11 | 0.16 | -0.05 | 0.03 | 0.08 | 0.06 | 0.02 | -0.11 | -0.03 | -0.03 | -0.14 | -0.14 |
| 24. SD annoyed | -0.09 | -0.06 | -0.01 | -0.02 | -0.01 | -*0.22* | -0.10 | *-0.21* | 0.17 | -0.005 | *0.20* | 0.11 | *-0.21* | **-0.29** | -0.13 | *-0.19* | *-0.20* |
| 25. SD lonely | 0.01 | 0.18 | -0.05 | -0.03 | 0.12 | -0.02 | 0.09 | *-0.20* | 0.01 | 0.02 | 0.08 | 0.02 | ***-0.34*** | ***-0.47*** | ***-0.35*** | ***-0.36*** | ***-0.37*** |
| 26. SD fight | <0.001 | -0.10 | -0.02 | 0.01 | -0.02 | **-0.24** | -0.06 | -0.17 | 0.07 | 0.10 | 0.06 | 0.09 | *-0.16* | -0.15 | -0.02 | -0.09 | -0.11 |
| 27. AR social | -0.03 | 0.01 | 0.06 | -0.11 | 0.005 | -0.07 | 0.08 | -0.07 | -0.10 | 0.08 | -0.14 | 0.08 | *-0.20* | -0.12 | -0.16 | -0.16 | -0.17 |
| 28. AR ease | -0.11 | 0.03 | -0.04 | -0.02 | 0.01 | 0.03 | 0.07 | -0.02 | 0.01 | 0.16 | 0.05 | 0.14 | *-0.21* | -0.12 | *-0.19* | *-0.19* | *-0.23* |
| 29. AR liked | -0.12 | 0.14 | -0.04 | -0.10 | 0.04 | 0.004 | -0.04 | -0.09 | 0.02 | 0.15 | 0.01 | 0.19 | **-0.28** | **-0.27** | -0.15 | -0.13 | -0.09 |
| 30. AR annoyed | -0.02 | -0.03 | <0.001 | -0.06 | -0.003 | -0.005 | 0.15 | -0.07 | *-0.21* | -0.03 | -0.02 | 0.08 | 0.01 | -0.001 | 0.05 | -0.008 | 0.02 |
| 31. AR lonely | 0.05 | 0.08 | -0.16 | -0.13 | 0.06 | 0.09 | 0.04 | 0.01 | -0.004 | -0.005 | 0.02 | 0.02 | *-0.22* | *-0.21* | -0.09 | -0.07 | -0.07 |
| 32. AR fight | -0.05 | 0.09 | -0.02 | -0.08 | -0.16 | 0.15 | -0.05 | -0.07 | -0.16 | -0.12 | 0.01 | **0.29** | -0.08 | -0.05 | -0.03 | -0.02 | 0.03 |
| 33. Age T1 | -0.002 | 0.14 | 0.002 | -0.11 | 0.07 | 0.04 | 0.01 | -0.01 | -0.12 | -0.04 | -0.001 | 0.13 | 0.03 | -0.09 | -0.07 | -0.12 | -0.12 |
| 34. Sex | -0.07 | -0.14 | 0.04 | -0.07 | -0.08 | -0.16 | -0.06 | -0.07 | -0.01 | -0.05 | 0.08 | -0.06 | 0.14 | 0.11 | -0.01 | 0.03 | -0.02 |
| 35. SES T1 | -0.03 | **0.28** | *0.21* | 0.02 | *-0.22* | -0.03 | -0.11 | -0.04 | 0.02 | -0.07 | 0.02 | -0.05 | 0.16 | 0.14 | 0.08 | 0.11 | 0.12 |
| 36. IQ T1 | -0.05 | 0.14 | -0.008 | -0.08 | -0.09 | -0.08 | -0.08 | -0.09 | -0.01 | -0.11 | 0.19 | -0.19 | 0.15 | 0.12 | -0.03 | 0.11 | 0.06 |

**Table S5-continued.** Bivariate correlations between the variables included in the SEM model. Correlations are computed on raw data (n=83-122).

|  | 18 | 19 | 20 | 21 | 22 | 23 | 24 | 25 | 26 | 27 | 28 | 29 | 30 | 31 | 32 | 33 | 34 | 35 | 36 |
| --- | --- | --- | --- | --- | --- | --- | --- | --- | --- | --- | --- | --- | --- | --- | --- | --- | --- | --- | --- |
| 18. M lonely | — |  |  |  |  |  |  |  |  |  |  |  |  |  |  |  |  |  |  |
| 19. M annoyed | ***0.63*** | — |  |  |  |  |  |  |  |  |  |  |  |  |  |  |  |  |  |
| 20. M fight | ***0.53*** | ***0.79*** | — |  |  |  |  |  |  |  |  |  |  |  |  |  |  |  |  |
| 21. SD social | 0.01 | -0.09 | *-0.19* | — |  |  |  |  |  |  |  |  |  |  |  |  |  |  |  |
| 22. SD ease | -0.03 | -0.15 | -0.17 | ***0.75*** | — |  |  |  |  |  |  |  |  |  |  |  |  |  |  |
| 23. SD liked | -0.01 | -0.13 | -0.13 | ***0.73*** | ***0.86*** | — |  |  |  |  |  |  |  |  |  |  |  |  |  |
| 24. SD annoyed | **0.25** | ***0.49*** | 0.16 | ***0.49*** | ***0.47*** | -0.14 | — |  |  |  |  |  |  |  |  |  |  |  |  |
| 25. SD lonely | ***0.65*** | **0.26** | 0.03 | ***0.46*** | *0.32* | -0.08 | ***0.52*** | — |  |  |  |  |  |  |  |  |  |  |  |
| 26. SD fight | **0.24** | ***0.42*** | ***0.42*** | **0.29** | **0.28** | -0.12 | ***0.65*** | ***0.37*** | — |  |  |  |  |  |  |  |  |  |  |
| 27. AR social | 0.08 | -0.13 | -0.11 | -0.01 | 0.03 | 0.01 | -0.13 | 0.03 | -0.03 | — |  |  |  |  |  |  |  |  |  |
| 28. AR ease | <0.001 | -0.07 | -0.11 | 0.13 | 0.12 | 0.14 | -0.01 | 0.07 | -0.001 | ***0.56*** | — |  |  |  |  |  |  |  |  |
| 29. AR liked | 0.03 | -0.01 | -0.10 | -0.02 | -0.06 | -0.06 | 0.02 | 0.03 | -0.01 | ***0.52*** | ***0.45*** | — |  |  |  |  |  |  |  |
| 30. AR annoyed | **0.29** | **0.27** | **0.26** | -0.17 | *-0.21* | -0.14 | 0.01 | *0.21* | 0.12 | 0.05 | 0.06 | 0.12 | — |  |  |  |  |  |  |
| 31. AR lonely | ***0.33*** | 0.05 | 0.07 | -0.12 | -0.12 | -0.08 | -0.08 | *0.21* | -0.006 | ***0.34*** | ***0.30*** | ***0.36*** | *0.18* | — |  |  |  |  |  |
| 32. AR fight | 0.16 | 0.19 | **0.28** | -0.11 | *-0.22* | -0.12 | -0.002 | 0.12 | 0.11 | 0.005 | 0.01 | 0.11 | **0.29** | **0.23** | — |  |  |  |  |
| 33. Age T1 | 0.12 | 0.03 | -0.04 | -0.07 | -0.11 | -0.08 | 0.03 | 0.12 | 0.02 | -0.03 | -0.08 | -0.004 | 0.09 | 0.06 | 0.09 | — |  |  |  |
| 34. Sex | -0.06 | 0.01 | 0.06 | -0.01 | 0.08 | 0.05 | -0.05 | -0.08 | -0.04 | -0.14 | *-0.20* | *-0.21* | 0.05 | -0.04 | -0.02 | -0.001 | — |  |  |
| 35. SES T1 | 0.02 | 0.03 | 0.001 | 0.13 | -0.03 | 0.03 | -0.01 | 0.08 | -0.03 | -0.02 | -0.05 | 0.02 | 0.11 | -0.05 | 0.15 | 0.05 | 0.12 | — |  |
| 36. IQ T1 | 0.12 | 0.13 | 0.01 | 0.07 | -0.12 | -0.16 | 0.03 | 0.12 | -0.04 | -0.13 | *-0.18* | 0.005 | 0.14 | -0.04 | 0.08 | 0.17 | 0.16 | **0.34** | — |

**Note.** Significant coefficients are indicated in bold for **p<0.01,** and in italics *p<0.05 and bold and italics for* ***p<0.001***. Sex was indexed 0=Female, 1=Male.

M-mean, SD- standard deviation, AR-autocorrelation, SES- socio-economic status, CBCL-Child Behavior Checklist, TRF-Teacher’s Report Form, GVSG-Groningen Social Behavior Questionnaire

*Daily diary items abbreviations*: social-‘I was social’ ; ease-‘I was at ease with others’; liked-‘I felt that others liked me’; annoyed-‘I felt that others were annoyed by me’; lonely-‘I felt lonely’; fight-‘I had a fight’

## **Table S6.** Correlation coefficients among latent factors describing parental, peer, and teacher affection and rejection during adolescence, from the CFA model run in Step 3 of the analysis.

|  | 1. | 2. | 3. | 4. | 5. |
| --- | --- | --- | --- | --- | --- |
| 1.Parental affection | - |  |  |  |  |
| 2.Parental rejection | -0.33 | - |  |  |  |
| 3.Teacher affection | 0.43 | **-0.71** | - |  |  |
| 4.Peer affection | 0.49 | -0.20 | **0.56** | - |  |
| 5.Peer rejection (TRF) | -0.18 | 0.49 | -0.21 | -0.003 | - |
| 6. Peer rejection (CBCL) | 0.06 | 0.34 | -0.22 | 0.34 | 0.12 |

**Note.** Significant coefficients are indicated in bold for **p<0.01,** and in italics for *p<0.05*. CFA is based on maximum likelihood with robust standard error estimation (MLR), sample size n=122.

CBCL-Child Behavior Checklist, TRF-Teacher’s Report Form

# Sensitivity analysis results

**Table S7.** Standardized regression coefficients derived from structural equation modeling (SEM) in which interpersonal functioning during young adulthood assessed at macro- and micro- level was predicted by the factors scores representing self and other reported parental, peer and teacher affection and rejection during adolescence, after the inclusion of covariates (sex, SES, IQ, and age at T1). In the right part of the table, the correlations coefficients from the SEM among the latent factors describing macro- and micro- level interpersonal functioning during young adulthood, after the inclusion of covariates (sex, SES, IQ, and age at T1), are presented.

|  | **Parental affection** | | **Parental rejection** | | **Teacher affection** | | **Peer affection** | | **Peer rejection- parent report** | | **Peer rejection- teacher report** | | **Age** | |
| --- | --- | --- | --- | --- | --- | --- | --- | --- | --- | --- | --- | --- | --- | --- |
|  | β (95% CI) | *p* | β (95% CI) | *p* | β (95% CI) | *p* | β (95% CI) | *p* | β (95% CI) | *p* | β (95% CI) | *p* | β (95% CI) | *p* |
| 1.Interpersonal functioning (GVSG) | 0.02  (-0.26 to 0.32) | 0.842 | 0.14  (-0.24 to 0.58) | 0.429 | -0.01  (-0.45 to 0.43) | 0.952 | 0.27  (-0.05 to 0.67) | 0.09 | -0.04  (-0.34 to 0.24) | 0.735 | -0.27  (-0.56 to -0.06) | *0.015* | -0.05  (-0.59 to 0.33) | 0.573 |
| 2.Mean positive daily social experiences | 0.06  (-0.16 to 0.30) | 0.547 | 0.17  (-0.20 to 0.56) | 0.357 | 0.04  (-0.31 to 0.40) | 0.796 | 0.03  (-0.27 to 0.34) | 0.844 | -0.06  (-0.31 to 0.17) | 0.585 | -0.13  (-0.31 to 0.04) | 0.129 | -0.13  (-0.70 to 0.13) | 0.176 |
| 3.Mean negative daily social experiences | -0.10  (-0.38 to 0.14) | 0.388 | -0.28  (-0.68 to 0.05) | 0.09 | 0.02  (-0.33 to 0.38) | 0.895 | -0.11  (-0.43 to 0.19) | 0.452 | 0.02  (-0.27 to 0.32) | 0.867 | 0.43  (0.21 to 0.74) | ***<0.001*** | -0.04  (-0.49 to 0.29) | 0.617 |
| 4.Variability positive daily social experiences | 0.07  (-0.17 to 0.31) | 0.553 | -0.05  (-0.52 to 0.40) | 0.809 | -0.12  (-0.60 to 0.35) | 0.603 | -0.01  (-0.52 to 0.48) | 0.945 | -0.1  (-0.24 to 0.40) | 0.959 | 0.07  (-0.15 to 0.30) | 0.511 | -0.09  (-0.60 to 0.21) | 0.344 |
| 5.Variability negative daily social experiences | -0.07  (-0.37 to 0.22) | 0.610 | -0.37  (-0.87 to 0.06) | 0.088 | -0.30  (-0.78 to 0.12) | 0.153 | -0.02  (-0.54 to 0.49) | 0.918 | 0.10  (-0.37 to 0.52) | 0.587 | 0.30  (0.06 to 0.59) | *0.01* | 0.02  (-0.37 to 0.49) | 0.792 |
| 6.Inertia positive daily social experiences | -0.11  (-0.40 to 0.17) | 0.430 | -0.32  (-0.75 to 0.06) | 0.095 | -0.21  (-0.59 to 0.14) | 0.238 | 0.15  (-0.25 to 0.57) | 0.441 | 0.16  (-0.20 to 0.55) | 0.362 | 0.12  (-0.13 to 0.39) | 0.320 | -0.04  (-0.50 to 0.28) | 0.584 |
| 7.Inertia negative daily social experiences | 0.01  (-0.35 to 0.37) | 0.951 | -0.32  (-0.99 to 0.24) | 0.238 | -0.21  (-0.78 to 0.28) | 0.367 | -0.002  (-0.58 to 0.57) | 0.992 | -0.30  (-0.86 to 0.18) | 0.208 | 0.25  (-0.15 to 0.72) | 0.204 | 0.08  (-0.34 to 0.77) | 0.450 |
| **Robust model fit indices** | | | | | | | | | | | | | | |
| **Chi square (df)** | 444.315 (262) | | | | | | | | | | | | | |
| **RMSEA** | 0.07 | | | | | | | | | | | | | |
| **CFI** | 0.90 | | | | | | | | | | | | | |
| **SRMR** | 0.06 | | | | | | | | | | | | | |

**Note.** Significant effects are marked in bold and italics for ***p<0.001,*** in bold for **p<0.01,** in italics for *p<0.05.* SEM are based on maximum likelihood with robust standard error estimation (MLR), sample size n=122.

**Table S7-continued.** Standardized regression coefficients derived from structural equation modeling (SEM) in which interpersonal functioning during young adulthood assessed at macro- and micro- level was predicted by the factor scores representing self and other reported parental, peer and teacher affection and rejection across adolescence, after the inclusion of covariates (sex, SES, IQ, and age at T1). In the right part of the table, the correlations coefficients from the SEM among the latent factors describing macro- and micro- level interpersonal functioning during young adulthood, after the inclusion of covariates (sex, SES, IQ, and age at T1), are presented.

|  | **Sex** | | **SES** | | **IQ** | |  |  |  |  |  |  |  |
| --- | --- | --- | --- | --- | --- | --- | --- | --- | --- | --- | --- | --- | --- |
|  | β (95% CI) | *p* | β (95% CI) | *p* | β (95% CI) | *p* | 1. | 2. | 3. | 4. | 5. | 6. | 7. |
| 1.Interpersonal functioning (GVSG) | 0.15  (-0.09 to 0.81) | 0.121 | 0.12  (-0.11 to 0.52) | 0.211 | 0.11  (-0.005 to 0.02) | 0.214 | - |  |  |  |  |  |  |
| 2.Mean level positive daily social experiences | -0.01  (-0.41 to 0.33) | 0.839 | 0.07  (-0.19 to 0.41) | 0.479 | 0.04  (-0.008 to 0.01) | 0.590 | ***0.58*** | - |  |  |  |  |  |
| 3.Mean level negative daily social experiences | -0.02  (-0.47 to 0.38) | 0.842 | 0.03  (-0.25 to 0.37) | 0.719 | 0.11  (-0.006 to 0.02) | 0.240 | -0.15 | -0.14 | - |  |  |  |  |
| 4.Variability positive daily social experiences | 0.07  (-0.22 to 0.52) | 0.434 | 0.03  (-0.18 to 0.29) | 0.653 | -0.12  (-0.02 to 0.009) | 0.335 | -0.10 | -0.15 | -0.16 | - |  |  |  |
| 5.Variability negative daily social experiences | -0.10  (-0.67 to 0.21) | 0.316 | -0.004  (-0.32 to 0.31) | 0.973 | 0.03  (-0.01 to 0.02) | 0.836 | **-0.35** | **-0.22** | 0.32 | ***0.60*** | - |  |  |
| 6.Inertia positive daily social experiences | -0.27  (-1.14 to -0.05) | *0.032* | 0.08  (-0.14 to 0.40) | 0.352 | -0.11  (-0.02 to 0.009) | 0.358 | **-0.34** | *-0.23* | -0.17 | 0.03 | -0.15 | - |  |
| 7.Inertia negative daily social experiences | -0.05  (-0.73 to 0.46) | 0.664 | 0.15  (-0.17 to 0.67) | 0.244 | 0.04  (-0.01 to 0.02) | 0.729 | -0.24 | 0.04 | *0.43* | *-0.41* | 0.03 | 0.48 | - |
|  |  |  |  |  |  |  |  |  |  |  |  |  |  |

**Note.** Significant effects are marked in bold and italics for ***p<0.001,*** in bold for **p<0.01,** in italics for *p<0.05.* SEM are based on maximum likelihood with robust standard error estimation (MLR), sample size n=122.

# Post hoc analysis results

**Table S8.** Standardized regression coefficients derived from structural equation modeling (SEM) in which interpersonal functioning during young adulthood assessed at macro- and micro-level was predicted by the factor scores describing self and other reported parental, peer and teacher affection and rejection separately at T1 and T3.

|  | **Parental affection T1** | | **Parental affection T3** | | **Parental rejection T1** | | **Parental rejection T3** | | **Teacher affection T1** | | **Teacher affection T3** | | **Peer affection T1** | |
| --- | --- | --- | --- | --- | --- | --- | --- | --- | --- | --- | --- | --- | --- | --- |
|  | β (95% CI) | *p* | β (95% CI) | *p* | β (95% CI) | *p* | β (95% CI) | *p* | β (95% CI) | *p* | β (95% CI) | *p* | β (95% CI) | *p* |
| 1.Interpersonal functioning (GVSG) | -0.003  (-0.30 to 0.29) | 0.981 | 0.06  (-0.20 to 0.36) | 0.599 | 0.02  (-0.31 to 0.39) | 0.839 | -0.1  (-0.37 to 0.34) | 0.935 | -0.19  (-0.68 to 0.17) | 0.250 | -0.07  (-0.40 to 0.22) | 0.570 | 0.15  (-0.22 to 0.64) | 0.352 |
| 2.Mean positive daily social experiences | 0.10  (-0.11 to 0.37) | 0.311 | 0.001  (-0.27 to 0.27) | 0.995 | -0.1  (-0.30 to 0.27) | 0.928 | 0.13  (-0.11 to 0.44) | 0.248 | -0.18  (-0.71 to 0.27) | 0.385 | 0.17  (-0.07 to 0.48) | 0.153 | 0.009  (-0.43 to 0.45) | 0.959 |
| 3.Mean negative daily social experiences | -0.07  (-0.39 to 0.19) | 0.489 | -0.21  (-0.56 to -0.009) | *0.04* | -0.03  (-0.37 to 0.27) | 0.757 | -0.14  (-0.47 to 0.06) | 0.133 | 0.05  (-0.24 to 0.39) | 0.648 | -0.13  (0.43 to 0.08) | 0.176 | -0.10  (-0.67 to 0.36) | 0.557 |
| 4.Variability positive daily social experiences | 0.03  (-0.18 to 0.26) | 0.721 | 0.07  (-0.25 to 0.41) | 0.635 | 0.02  (-0.22 to 0.29) | 0.800 | 0.02  (-0.29 to 0.35) | 0.953 | 0.04  (-0.30 to 0.41) | 0.767 | -0.17  (-0.57 to 0.17) | 0.294 | 0.25  (0.003 to 0.62) | *0.04* |
| 5.Variability negative daily social experiences | -0.06  (-0.29 to 0.11) | 0.406 | -0.13  (-0.44 to 0.11) | 0.258 | -0.03  (-0.32 to 0.24) | 0.775 | -0.16  (-0.46 to 0.04) | 0.112 | 0.21  (-0.02 to 0.57) | 0.07 | -0.32  (-0.72 to -0.08) | *0.01* | -0.05  (-0.42 to 0.27) | 0.677 |
| 6.Inertia positive daily social experiences | -0.007  (-0.36 to 0.34) | 0.962 | 0.10  (-0.20 to 0.44) | 0.453 | -0.03  (-0.38 to 0.30) | 0.821 | -0.10  (-0.49 to 0.22) | 0.472 | -0.17  (-0.65 to 0.24) | 0.369 | 0.15  (-0.17 to 0.54) | 0.304 | 0.09  (-0.42 to 0.66) | 0.666 |
| 7.Inertia negative daily social experiences | 0.04  (-0.34 to 0.48) | 0.752 | 0.01  (-0.35 to 0.39) | 0.918 | -0.18  (-0.67 to 0.16) | 0.226 | -0.17  (-0.68 to 0.20) | 0.291 | -0.12  (-0.75 to 0.42) | 0.577 | 0.03  (-0.37 to 0.47) | 0.826 | -0.17  (-0.85 to 0.33) | 0.383 |
| **Robust model fit indices** | | | | | | | | | | | | | | |
| **Chi square (df)** | 471.71 (287) | | | | | | | | | | | | | |
| **RMSEA** | 0.08 | | | | | | | | | | | | | |
| **CFI** | 0.87 | | | | | | | | | | | | | |
| **SRMR** | 0.07 | | | | | | | | | | | | | |

**Note.** Significant effects are marked in bold and italics for ***p<0.001,*** in bold for **p<0.01,** in italics for *p<0.05.* SEM are based on maximum likelihood with robust standard error estimation (MLR), sample size n=122.

**Table S8-continued.** Standardized regression coefficients derived from structural equation modeling (SEM) in which interpersonal functioning during young adulthood assessed at macro- and micro-level was predicted by the factor scores representing self and other reported parental, peer and teacher affection and rejection separately at T1 and T3. In the right part of the table, the correlations coefficients from the SEM among the latent factors describing macro- and micro- level interpersonal functioning during young adulthood are presented.

|  | **Peer affection T3** | | **Peer rejection**  **teacher report T1** | | **Peer rejection**  **teacher report T3** | | **Peer rejection**  **parent report T1** | | **Peer rejection**  **parent report T3** | | *1.* | *2.* | *3.* | *4.* | *5.* | *6.* | *7.* |
| --- | --- | --- | --- | --- | --- | --- | --- | --- | --- | --- | --- | --- | --- | --- | --- | --- | --- |
|  | β (95% CI) | *p* | β (95% CI) | *p* | β (95% CI) | *p* | β (95% CI) | *p* | β (95% CI) | *p* |  |  |  |  |  |  |  |
| 1.Interpersonal functioning (GVSG) | 0.30  (0.04 to 0.74) | *0.02* | 0.16  (-0.09 to 0.49) | 0.18 | -0.30  (-0.68 to -0.04) | *0.02* | -0.04  (-0.38 to 0.26) | 0.723 | -0.003  (-0.43 to 0.43) | 0.986 | ***-*** |  |  |  |  |  |  |
| 2.Mean positive daily social experiences | 0.18  (-0.13 to 0.56) | 0.222 | -0.002  (-0.26 to 0.26) | 0.986 | -0.04  (-0.32 to 0.23) | 0.728 | 0.14  (-0.11 to 0.43) | 0.262 | -0.06  (-0.45 to 0.27) | 0.633 | ***0.55*** | - |  |  |  |  |  |
| 3.Mean negative daily social experiences | -0.03  (-0.45 to 0.36) | 0.832 | 0.45  (0.34 to 0.88) | ***<0.001*** | 0.29  (0.04 to 0.70) | *0.02* | 0.07  (-0.23 to 0.42) | 0.571 | -0.17  (-0.71 to 0.17) | 0.226 | -0.20 | -0.17 | - |  |  |  |  |
| 4.Variability positive daily social experiences | -0.15  (-0.71 to 0.35) | 0.507 | 0.06  (-0.15 to 0.30) | 0.536 | 0.01  (-0.25 to 0.28) | 0.912 | 0.06  (-0.38 to 0.52) | 0.762 | -0.03  (-0.45 to 0.36) | 0.826 | -0.05 | -0.16 | -0.11 | - |  |  |  |
| 5.Variability negative daily social experiences | -0.03  (-0.53 to 0.44) | 0.855 | 0.16  (-0.05 to 0.43) | 0.119 | 0.24  (0.04 to 0.52) | *0.01* | 0.14  (-0.15 to 0.49) | 0.246 | 0.01  (-0.26 to 0.29) | 0.920 | -0.28 | **-0.25** | 0.25 | ***0.53*** | - |  |  |
| 6.Inertia positive daily social experiences | 0.06  (-0.36 to 0.50) | 0.744 | 0.01  (-0.39 to 0.43) | 0.918 | 0.26  (-0.01 to 0.60) | 0.06 | -0.07  (-0.48 to 0.32) | 0.695 | -0.07  (-0.48 to 0.27) | 0.598 | *-0.42* | -0.13 | -0.05 | -0.11 | 0.03 | - |  |
| 7.Inertia negative daily social experiences | -0.04  (-0.70 to 0.57) | 0.845 | 0.23  (-0.18 to 0.82) | 0.217 | 0.40  (-0.23 to 1.26) | 0.173 | -0.25  (-0.89 to 0.21) | 0.234 | -0.24  (-1.27 to 0.51) | 0.408 | -0.51 | 0.10 | 0.41 | -0.50 | -0.01 | 0.54 | - |

**Note:** Significant effects are marked in bold and italics for ***p<0.001,*** in bold for **p<0.01,** in italics for *p<0.05.* SEM are based on maximum likelihood with robust standard error estimation (MLR), sample size n=122.

# References

Achenbach, T. (2001). *Manual for the ASEBA School-Age Forms & Profiles.* University of Vermont.

Markus, M. T., Lindhout, I. E., Boer, F., Hoogendijk, T. H. G., & Arrindell, W. A. (2003). Factors of perceived parental rearing styles: The EMBU-C examined in a sample of Dutch primary school children. *Personality and Individual Differences*, *34*(3), 503–519. https://doi.org/10.1016/S0191-8869(02)00090-9

Ormel, J., Lindenberg, S., Steverink, N., & Vonkorff, M. (1997). Quality of life and social production functions: A framework for understanding health effects. *Social Science and Medicine*, *45*(7), 1051–1063. https://doi.org/10.1016/S0277-9536(97)00032-4

Tilton-Weaver, L., Kerr, M., Pakalniskeine, V., Tokic, A., Salihovic, S., & Stattin, H. (2010). Open up or close down: How do parental reactions affect youth information management? *Journal of Adolescence*, *33*(2), 333–346. https://doi.org/10.1016/j.adolescence.2009.07.011
